# BMECs Ameliorate High Glucose-Induced Morphological Aberrations and Synaptic Dysfunction via VEGF-Mediated Modulation of Glucose Uptake in Cortical Neurons

**DOI:** 10.1007/s10571-023-01366-0

**Published:** 2023-07-07

**Authors:** Yu-Qi Huang, Xiao Gu, Xiao Chen, Yi-Ting Du, Bin-Chi Chen, Feng-Yan Sun

**Affiliations:** 1 Department of Neurobiology and Research Institute for Aging and Medicine, School of Basic Medical Sciences, Shanghai Medical College, Fudan University, 138 Yi-Xue-Yuan Road, Shanghai, 200032 People’s Republic of China; 2 National Clinical Research Center for Aging and Medicine, Shanghai Medical College, Fudan University, Shanghai, 200032 People’s Republic of China; 3 Shanghai Key Laboratory of Bioactive Small Molecules, School of Basic Medical Sciences, Institute of Biomedical Sciences, Shanghai Medical College, Fudan University, Shanghai, 200032 People’s Republic of China

**Keywords:** Diabetes, Neuritic dystrophy, Synapsis formation, Brain microvascular endothelial cell, VEGF, Glucose uptake

## Abstract

It has been demonstrated that diabetes cause neurite degeneration in the brain and cognitive impairment and neurovascular interactions are crucial for maintaining brain function. However, the role of vascular endothelial cells in neurite outgrowth and synaptic formation in diabetic brain is still unclear. Therefore, present study investigated effects of brain microvascular endothelial cells (BMECs) on high glucose (HG)-induced neuritic dystrophy using a coculture model of BMECs with neurons. Multiple immunofluorescence labelling and western blot analysis were used to detect neurite outgrowth and synapsis formation, and living cell imaging was used to detect uptake function of neuronal glucose transporters. We found cocultured with BMECs significantly reduced HG-induced inhibition of neurites outgrowth (including length and branch formation) and delayed presynaptic and postsynaptic development, as well as reduction of neuronal glucose uptake capacity, which was prevented by pre-treatment with SU1498, a vascular endothelial growth factor (VEGF) receptor antagonist. To analyse the possible mechanism, we collected BMECs cultured condition medium (B-CM) to treat the neurons under HG culture condition. The results showed that B-CM showed the same effects as BMEC on HG-treated neurons. Furthermore, we observed VEGF administration could ameliorate HG-induced neuronal morphology aberrations. Putting together, present results suggest that cerebral microvascular endothelial cells protect against hyperglycaemia-induced neuritic dystrophy and restorate neuronal glucose uptake capacity by activation of VEGF receptors and endothelial VEGF release. This result help us to understand important roles of neurovascular coupling in pathogenesis of diabetic brain, providing a new strategy to study therapy or prevention for diabetic dementia.

**Graphical Abstract:**

Hyperglycaemia induced inhibition of neuronal glucose uptake and impaired to neuritic outgrowth and synaptogenesis. Cocultured with BMECs/B-CM and VEGF treatment protected HG-induced inhibition of glucose uptake and neuritic outgrowth and synaptogenesis, which was antagonized by blockade of VEGF receptors. Reduction of glucose uptake may further deteriorate impairment of neurites outgrowth and synaptogenesis.
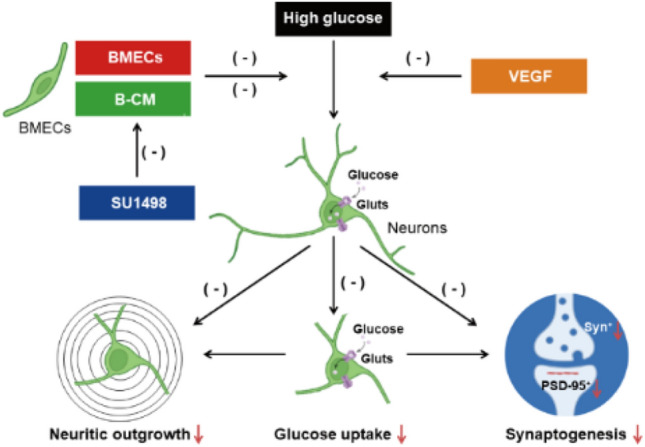

**Supplementary Information:**

The online version contains supplementary material available at 10.1007/s10571-023-01366-0.

## Introduction

It has been found that diabetes not only damages peripheral nerves but also leads to central neuropathy and cognitive dysfunction (diabetic encephalopathy) (Reske-Nielsen and Lundbaek 1968; Reske-Nielsen et al. 1966, 1970). Postmortem results have revealed the occurrence of neuroaxonal dystrophy (NAD), a type of axonal lesion, in the sympathetic ganglia of diabetic patients (Duchen et al. 1980). NAD is characterized by significant distal axon enlargement accompanied by a specific reduction in neuronal density that affects the connections between neurons (Schmidt et al. 1993). Moreover, diabetic rats show neuropathological features consistent with those of diabetic patients (Schmidt 2001; Schmidt et al. 2003, 2008a, b), such as dendritic swelling and reductions in neurite length and branching(Schmidt et al. 2000). The previous research results of our group have further confirmed that diabetic rats exhibit damage to cortical neuronal processes in the brain and cognitive impairment (Zhao et al. 2013). In addition, in type 2 diabetes (T2D) patients with persistent hyperglycaemia, there is a significant increase in lesion volume in white matter region (Saczynski et al. 2009), accompanied by hippocampal and amygdala atrophy (Cheng et al. 2012), which may be due to decreased neurogenesis and increased neuronal death (Pugazhenthi et al. 2017). The white matter in the brain contributes to the formation of synapses and the transmission of information between neural circuits; therefore, white matter damage can lead to loss of synapses, which in turn can lead to a decline in learning and memory (Alexander et al. 2018).

Neurons are the most important structural and functional units of the central nervous system, and neuronal transmission mainly depends on dendrites; thus, changes in dendrite size or structure affect the function of neurons (Alexander et al. 2018). Although early human studies failed to identify dendritic swelling in the brains of diabetic patients, they found a small decrease in neuronal density (~ 14%) in the prevertebral sympathetic ganglia of diabetic patients (Schmidt et al. 1993). Due to the large number of neurons, it is impossible to quantitatively analyse the damage to dendrites in the brains of diabetic patients. Therefore, in this study, days in vitro (DIV)7 and DIV14 cortical neurons were treated with 45 mM glucose in vitro (Russell et al. 2002) and then detected by microtubule-associated protein 2 (Map2) immunofluorescence staining. To quantitatively analyse neuronal dendrites, Sholl analysis was used to analyse the length and complexity of neuronal processes. In Sholl analysis, a group of isometric concentric circles is superimposed on the cell body of a reconstructed neuron, the numbers of branches intersecting each circle is calculated, and the branching patterns of neuronal dendrites and axons are determined to quantitatively characterize the morphological characteristics of the neuron (Yang et al. 2016). In addition, the size of a neuron’s dendrites affects the numbers of synapses it forms with axon terminals.

In the central nervous system, neurons connect with each other through special structures called synapses to form neural circuits (Südhof 2021). Synapses are divided into electrical synapses and chemical synapses, among which chemical synapses play a major role in neuronal transmission. Therefore, in neurological disorders, cognitive deficits are often associated with synaptic abnormalities, such as abnormal changes in synaptic density in autism (Coghlan et al. 2012), schizophrenia (Sellgren et al. 2019), and Alzheimer disease (AD) (Scheff and Price 2006). However, during chemical synaptic transmission, the exocytosis of synaptic vesicles rich in neurotransmitters at the presynaptic membrane and the expression of receptors on the postsynaptic membrane can affect the transmission of information (Uchigashima et al. 2016). The postsynaptic density (PSD) is located on the inner side of the postsynaptic membrane. Postsynaptic density-95 (PSD95) is one of the main components of the PSD (Cheng et al. 2006; Cho et al. 1992). PSD95 recruits NMDARs (N-methyl-D-aspartate receptors) and AMPARs (α-amino-3-hydroxy-5-methyl-4-isoxazole propionic acid receptors) and is involved in the maturation of excitatory synapses and the formation of dendritic spines (Cho et al. 1992; Coley and Gao 2018). Furthermore, PSD95 can affect the dendritic structure of hippocampal neurons during their development (Jeyifous et al. 2016; Mardones et al. 2019). In addition, a reduction in the number of PSD95-positive postsynaptic dendrites was observed on pyramidal neurons in the cortex and hippocampal CA1 region in diabetic mice (Yan et al. 2016). Therefore, in this study, the distribution of presynaptic vesicles and PSD95 and changes of the level of labeled proteins in neurons were assessed by immunofluorescence before and after treatment with high glucose to explore the effect of high glucose on synapse formation.

Unlike neurons cultured in vitro, neurons in the brain have close structural and functional connections with blood vessels (mainly endothelial cells), which constitute the neurovascular unit (NVU). A large numbers of studies have shown that vascular endothelial cells can also participate in neural plasticity in developing and adult brain, as well as the synthesis and secretion of vascular endothelial growth factor (VEGF) (Wu et al. 2017). In the central nervous system, VEGF not only promotes angiogenesis (Melincovici et al. 2018) but also plays a role in neuronal nutrition and neuroprotection (Storkebaum et al. 2004). However, the role of vascular endothelial cells in neurite damage in diabetic brain is not clear. Therefore, in this study, we constructed a direct contact coculture system comprising brain microvascular endothelial cells (BMECs), neurons and endothelial cell-conditioned culture medium and found that endothelial cells have a protective effect against neuronal process injury caused by high glucose via secreting VEGF.

The mechanisms underlying neuritic dystrophy and dysfunction in diabetic patients have not been identified, but impairment of glucose regulation in the brain may be one of them (de Ceballos and Köfalvi 2017). The brain is known to consume approximately a quarter of the body’s energy supply (Magistretti and Allaman 2018), with neurons consuming approximately 85% of the brain’s nutrients (Ashrafi et al. 2017; Dienel 2019; Engl and Attwell 2015). Approximately 95% of ATP, the main energy source, is produced via glucose metabolism (Magistretti and Allaman 2018). Glucose, a major nutrient, enters the brain from the periphery by crossing the blood‒brain barrier (BBB), a major component of which is endothelial cells (Devraj et al. 2011), via glucose transporter 1 (GLUT1) and is then transported to neurons via glucose transporters 3 (GLUT3) (Peng et al. 2021). In diabetic animals, downregulation of GLUT1 expression in the BBB reduces peripheral glucose entry into the brain (Pardridge et al. 1990). Similarly, patients with poorly controlled T2DM patients were found to have significantly lower levels of glucose than healthy subjects (Hwang et al. 2017). Therefore, in this experiment, after cortical neurons were treated with high glucose, 2-[N-(7-nitrobenz-2-oxa-1,3-diazol-4-yl) amino]-2-deoxy-D-glucose (2-NBDG) was used to monitor glucose uptake by neurons, and GLUT1 and GLUT3 protein levels were measured by western blotting to explore whether high glucose stimulation can impair neuronal glucose uptake in vitro. The results showed that endothelial cells restored the uptake capacity of neuronal glucose transporters under high-glucose conditions by releasing VEGF. The findings provide a new direction for the treatment of neuritic dystrophy and dysfunction in the brains of diabetic patients and delay the progression of neurodegeneration in diabetic patients.

## Materials and Methods

The approval of this study was granted by the Ethics Committee of Shanghai Medical College, Fudan University (No.20180302-108). Each of the experiments and data analysis described below were carried out in a randomized order by the experimenter blinded to the group.

### Primary Cortical Neuron Culture

Primary cortical neurons were prepared as previously described (Wu et al. 2016). Briefly, the cerebral cortices of rat E17 embryos were cut into small pieces and then treated with trypsin-EDTA for 10 min at 37 °C. The cells were seeded at a density of 0.8 × 10^6^ cells/ml in poly-l-lysine (0.01%)-coated dishes containing Dulbecco’s modified Eagle medium supplemented with 10% foetal bovine serum and 10 units/ml penicillin and streptomycin and incubated at 37 °C in 5% CO_2_ in a humidified 95% air atmosphere. After 1 h, the neurons were cultured in neurobasal medium supplemented with 2% B27, and the medium was changed every 2 to 3 days.

### Brain Microvascular Endothelial Cell Culture

BMECs were isolated from 1-day-old Sprague‒Dawley rats and cultured as described in our previous study (Wu et al. 2016). Briefly, cortical tissues were collected, the meninges were removed, and the tissues were cut into 1 mm^3^ pieces and digested with 1% type II collagenase in DMEM at 37 °C for 1 h. The mixtures were centrifuged at 500 rpm for 5 min. The precipitates were then collected and resuspended in 20% BSA. The mixtures were centrifuged again at 1000 rpm for 10 min. The pellets were resuspended in PBS, layered onto a Percoll gradient and centrifuged at 1000×*g* for 10 min at 4 °C. The cells were resuspended in EGM2-MV (containing 5 µg/ml puromycin on the first 2 days) supplemented with Supplement Mix (containing growth factors and 5% FBS) and seeded onto 2% gelatine/collagen-coated dishes. The cells were cultured at 37 °C in 5% CO_2_ for 1–2 weeks, subcultured and used for experiments after reaching 80–90% confluence.

### Coculture of Neurons with Endothelial Cells and Preparation of High-Glucose BMEC-Conditioned Medium

To mimic the NVU microenvironment under high-glucose conditions, we prepared direct endothelial cell-neuron coculture systems and indirect BMEC-conditioned medium (B-CM). BMECs were trypsinized with 0.25% trypsin-EDTA, seeded at a density of 0.4 × 10^6^ cells/ml in neurobasal medium containing 2% B27 supplement to DIV7 or DIV14 cultured neurons. Following 24 h of culture, the neurons were used for immunofluorescence. For western blotting, we established an indirect coculture model. Neurons were seeded in a 6-well plate coated with 0.01% poly-l-lysine, and inserts were placed in the 6-well plate. BMECs were seeded in the upper compartment of each insert, which was coated with 2% gelatine/collagen.

For the preparation of B-CM, BMECs were grown to 70–80% confluence and subsequently incubated in neurobasal medium containing 2% B27 supplement and 45 mM glucose for 6 h. Then, the supernatants were passed through a 0.22 μm filter and used as high-glucose B-CM under for subsequent in vitro studies.

For VEGF receptor-2 (VEGFR-2) antagonist SU1498 study, the neurons were preincubated in the presence of 10 µM VEGFR-2 inhibitor SU1498 (AG1498, MedChemExpress, New Jersey, America ) for 1 h prior. Then, the neurons were incubated in the medium containing normal glucose or B-CM with high glucose.

**Fig. 1:**
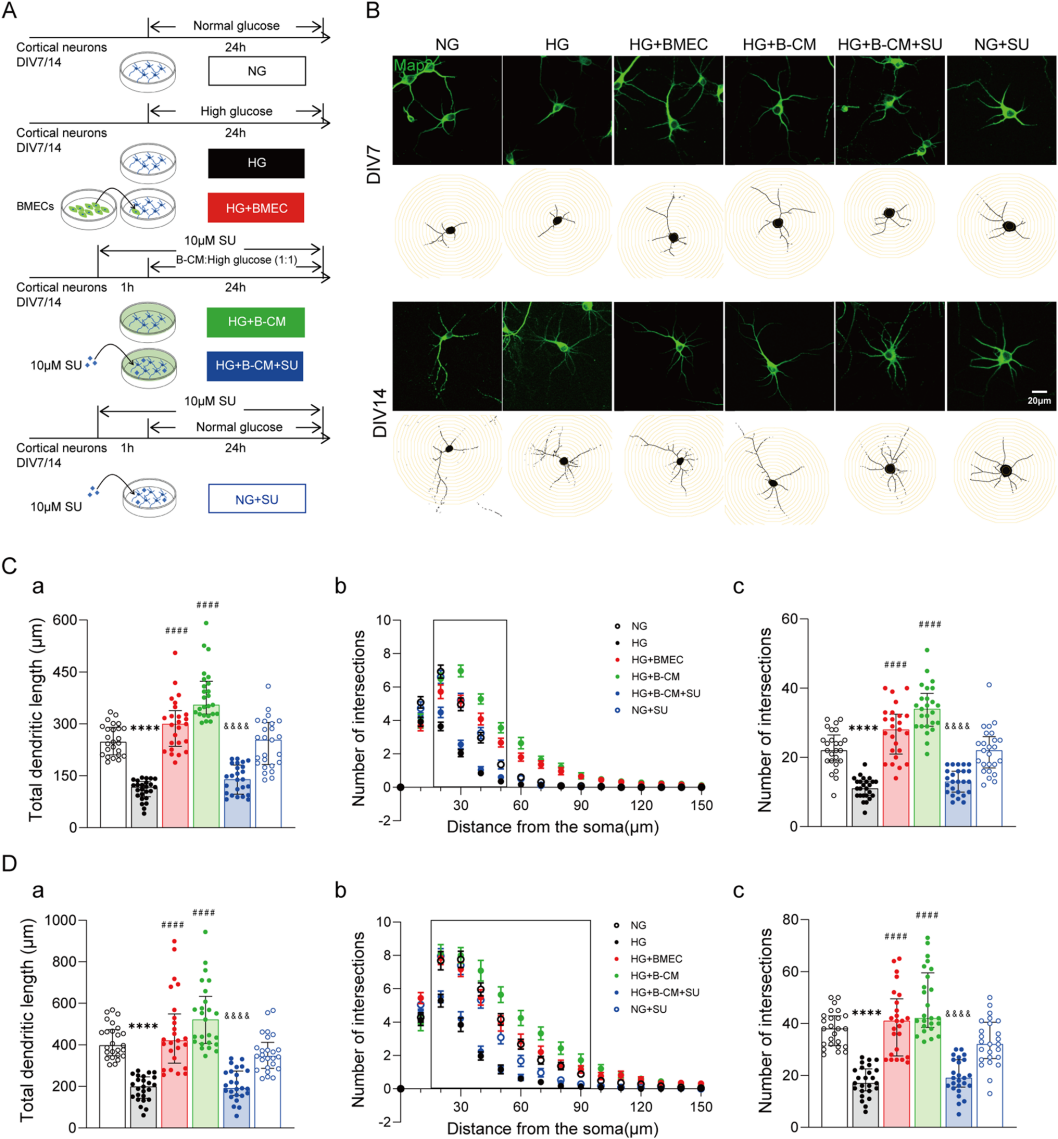
BMECs ameliorate the high glucose−induced reductions in neuritic growth of cortical neurons through activation of VEGF receptors. **A** Schematic of the experimental procedures. **B** Confocal images and illustrations of Sholl analysis of the dendrites of cortical neurons. Neuronal and dendrite morphology was assessed by Map2 staining. **C**–**D** Morphometric analysis of **a** the total length of dendrites and **b**−**c** Sholl analysis of dendrites of cortical neurons grown with BMECs and treated with B−CM or treated with B−CM with SU1498 (10 µM) under high−glucose conditions compared with neurons cultured with high glucose alone for 24 h. **b** Plots of the numbers of intersections as a function of distance from the soma. **c** Sholl analysis was further used to quantify the total numbers of process intersections. *n* = 25. ****p < 0.0001 vs. NG; ####*p* <0.0001 vs. HG; &&&&*p* <0.0001 vs. HG +B−CM. Bars: 20 μm. The data are presented as the median and interquartile range or mean ± SD. NG, neurons cultured alone and treated with 25 mM glucose; HG, neurons cultured alone and treated with 45 mM glucose; HG+BMEC, neurons grown with BMECs in 45 mM glucose; HG+B−CM, neurons treated with B−CM and 45 mM glucose; HG+B−CM+SU, neurons treated with B−CM, 45 mM glucose and 10 µM SU1498; NG+SU, neurons cultured with 10 µM SU1498 and treated with 25 mM glucose

All treatment protocols for each group were illustrated in the Fig. 1A.

### Immunofluorescent Staining

Cells were fixed with 4% paraformaldehyde and then blocked in PBS containing 10% FBS and 0.1% Triton X-100 for 1 h at 37 °C. Primary antibodies against PSD95 (mouse monoclonal antibody, 1:100, catalog #: MAB1596, PMID: 25,498,153, Sigma-Aldrich, St.Louis, USA), Map2 (mouse monoclonal antibody, 1:500, catalog #: M9942, PMID: 26,903,822, Sigma‒Aldrich, St.Louis, USA; rabbit polyclonal antibody, 1:500, catalog #: ab32454, Abcam, Cambridge, UK) and synapsin-1 (rabbit monoclonal antibody, 1:100, catalog #: ab254349, PMID: 9,539,796, Abcam, Cambridge, UK) were incubated with the cells overnight at 4 °C. Then, appropriate fluorescent secondary antibodies (A-21,202, A-21,207 or A-21,206, Life Technologies, California, USA) diluted 1:1000 were incubated with the cells for 1 h at 37 °C in the dark. Following DAPI staining, the cells were mounted and analysed under confocal laser scanning microscopy (SP8, Leica, Wetzlar, Germany).

### Neurite Analysis

After treatment, neurons were labelled by Map2 immunofluorescence, and images were captured by confocal microscopy (SP8, Leica, Wetzlar, Germany). The total length of neuronal dendrites (µm) was analysed by the Neuron J plug-in of ImageJ software. A Sholl analysis plug-in was used to analyse the complexity of neurites (the number of intersections). To determine the number of intersections of each neuron, concentric circles were drawn at 10-µm intervals, with the neuronal cell body as the centre of each circle, and the numbers of intersections between neurites and the concentric circles was determined.

### Western Blotting

For protein extraction, the cells were lysed with cell lysis buffer containing phosphatase inhibitors for 10 min at 4 °C. The extracts were centrifuged at 12,000×*g* at 4 °C for 20 min, and the supernatants were collected. The protein concentration of each sample was quantified using the bicinchoninic acid (BCA) assay. For western blotting, 20 µg of protein was separated on SDS polyacrylamide gels and transferred to PVDF membranes at 100 V in transfer buffer (25 mM Tris, 192 mM glycine, 20% (v/v) methanol) for 70 min at 4 °C. The membranes were blocked for 1 h at room temperature in Tris-buffered saline-Tween (TBS-T) containing 5% skim milk and incubated with primary antibodies: mouse anti-PSD95, rabbit anti-synapsin-I, mouse anti-GLUT1 (1:1000, catalog #: sc-377,228, PMID: 30,464,533, Santa Cruz, CA, USA), rabbit anti-GLUT3 (1:1000, catalog #: 400,062, PMID: 25,855,193, Sigma‒Aldrich, St. Louis, USA ), and β-actin (1:10,000; 3700; Cell Signaling Technology, MA, USA) in TBS-T. After incubation, the blots were incubated in horseradish peroxidase-conjugated anti-mouse or anti-rabbit IgG (1:3000, sc-516,102 or sc-2357, Santa Cruz, CA, USA) in TBS-T for 1 h at room temperature and imaged with the ChemiDoc Imaging System. Protein levels were quantified by measuring the density of each band using ImageJ software and then normalized to the β-actin level.

### Glucose Uptake Assay

Neurons were incubated in the presence of 2-NBDG (50 µM) (N13195, Invitrogen, California, USA) for 24 h at 37 °C and washed with ice-cold PBS to stop the reaction. The fluorescent signals were captured at 488 nm by time-lapse video microscopy according to the methods described for time-lapse microscopy ( AF6000, Leica, Wetzlar, Germany). The neurons were analysed in quintuplicate under each condition, and the images were analysed with ImageJ/Fiji software by setting a threshold and measuring the integrated density per frame.

### Statistical Analysis

Normal distribution and variance homogeneity were assessed for each dataset using the Shapiro–Wilk normality and the Levene test, respectively. When the assumptions of normal distribution and homogeneity of variance were met, parametric tests were performed, and the data was expressed as mean ± SD. Otherwise, the data was reported as median with interquartile range and analysed using non-parametric tests. One-way ANOVA was performed when multiple means were compared. Statistical analyses were performed using GraphPad Prism (GraphPad Prism 9.0; GraphPad Software Inc., La Jolla, CA) and SPSS. All experiments were independently repeated at least five times. The sample sizes were estimated based on previously pilot experiments and calculated using the software G-power. The detailed statistics is in Supplementary Material. *p* < 0.05 was considered significant.

## Results

### High Glucose Treatment Diminishes Dendritic Arborization and the Levels of PSD95 and Synapsin-I in Cortical Neurons

Hyperglycaemia is known to damage the central nervous system and cause pathological changes in nerve processes. Therefore, to establish a model of diabetes-induced neuronal process degeneration in vitro, DIV7 and DIV14 neurons were treated with high glucose (neurobasal medium containing 45 mM glucose) for 24 h based on previous literature (Russell et al. 2002). To evaluate the effect of hyperglycaemia on the dendritic morphology and synaptogenesis of cortical neurons, DIV7 and DIV14 cortical neurons were treated for 24 h with high glucose (HG, 45 mM D-glucose) and normal glucose (NG, 25 mM D-glucose). The dendritic morphology of each individual neuron was revealed by immunostaining with Map2, a dendritic marker (Fig. 1B). The Neuron J plug-in of ImageJ software and the Sholl Analysis plug-in were used to analyse process length and process complexity (i.e., the number of intersections), respectively (Fig. 1C–D). The following morphometric parameters were analysed: total dendritic length (Fig. 1C-a and D-a), numbers of intersections as a function of distance from the soma (Fig. 1C-b and D-b) and total numbers of intersections (Fig. 1C-c and D-c). Compared with NG treated group, neurons in HG treated group showed a widespread pathological morphology, with extremely short neurites. As shown in Fig. 1C-a and D-a, the average process lengths (µm) of DIV7 and DIV14 neurons in the HG group were 117.18 (89.22, 133.21) and 198.84 (143.43, 245.98), respectively, which were significantly lower than that in the NG group (*p*_DIV7_ <0.0001; *p*_DIV14_ <0.0001; Table 1). To analysis whether hyperglycaemia stimulation reduces the complexity of neuronal processes, the numbers of neuronal intersections were statistically analysed. As shown in Fig. 1C-b, the numbers of process intersections at 20, 30, 40 and 50 μm from the cell body were significantly lower for DIV7 neurons in the HG group than that in the NG group (*p*_20µm_ <0.0001; *p*_30µm_ <0.0001; *p*_40µm_ <0.001; *p*_50µm_ = 0.021; Supplementary Material). The numbers of process intersections of DIV14 neurons in the HG group were significantly decrease at 20, 30, 40, 50, 60 ,70, 80 and 90 μm from the cell body than that in the NG group (*p*_20µm_ =0.037; *p*_30µm_ <0.0001; *p*_40µm_ <0.0001; *p*_50µm_ <0.0001; *p*_60µm_ <0.0001; *p*_70µm_ =0.005; *p*_80µm_ = 0.001; *p*_90µm_ = 0.010; Supplementary Material). To further compare the influence of high glucose treatment on the morphological complexity of DIV7 and DIV14 neurons, the average numbers of process intersections were statistically analysed. As shown in Fig. 1C-c and D-c, the average numbers of process intersections for DIV7 and DIV14 neurons in the HG group were 11.00 (9.00, 13.00) and 17.00 (14.00, 22.50), respectively, which were significantly lower than that in the NG group (*p*_DIV7_ < 0.0001; *p*_DIV14_ < 0.0001; Table 1). In conclusion, high glucose stimulation can reduce the length and complexity of neurite processes of cultured neurons.

**Fig. 2:**
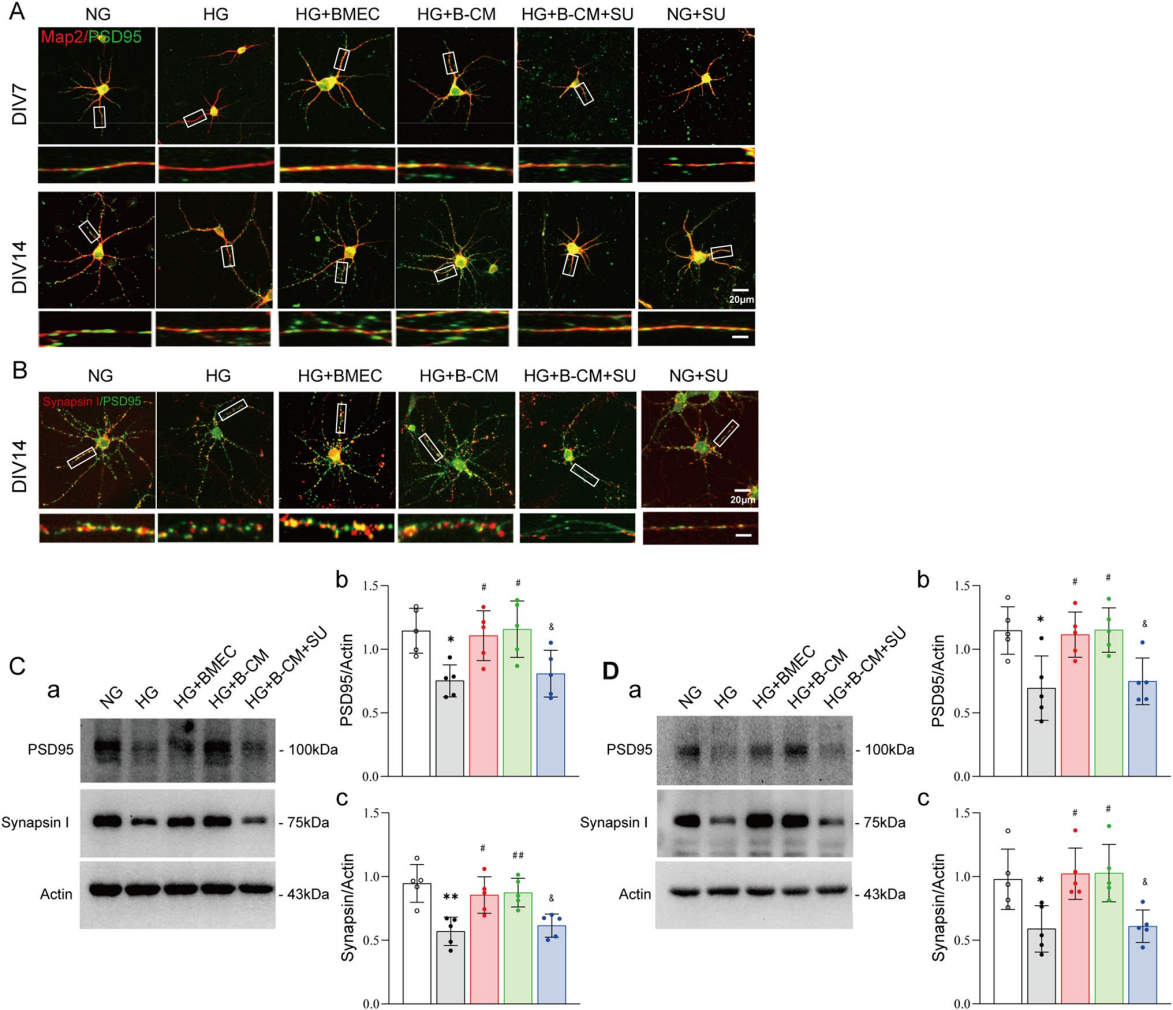
BMECs ameliorate the high glucose−induced reductions in synaptogenesis of cortical neurons through activation of VEGF receptors. **A**, **B** Representative images of immunostaining of PSD95 (green) and the dendritic marker protein Map2 (red) or PSD95 (green) and the general presynaptic marker protein Synapsin I (red) in neurons grown with BMECs and treated with B−CM or treated with B−CM with SU1498 (10 µM) under high−glucose conditions compared with neurons cultured with high glucose alone for 24 h. **C**, **D** Immunoblots for PSD95 and Synapsin I in cultured cortical neurons. Actin was used for normalization. *n* =5. *p < 0.05, **p < 0.01 vs. NG; #*p* < 0.05, ##*p* < 0.01vs HG; &*p* < 0.05 vs. HG +B−CM. Bars: 20 μm; magnification: 5 μm. The data are presented as mean ± SD. NG, neurons cultured alone and treated with 25 mM glucose; HG, neurons cultured alone and treated with 45 mM glucose; HG+BMEC, neurons grown with BMECs in 45 mM glucose; HG+B−CM, neurons treated with B−CM and 45 mM glucose; HG+B−CM+SU, neurons treated with B−CM, 45 mM glucose and 10 µM SU1498; NG+SU, neurons cultured with 10 µM SU1498 and treated with 25 mM glucose

Synapses serve as the structural basis for information transmission between neurons. PSD95 is closely related to neuronal process development, excitatory synaptic maturation and the formation of dendritic spines. Therefore, double immunofluorescence staining of PSD95 (green fluorescence) with Map2 (red fluorescence) or with synapsin I (red fluorescence), a presynaptic vesicle marker, was performed on DIV7 and DIV14 neurons stimulated by high glucose (Fig. 2A–B). The results showed that PSD95 positive (PSD95^+^) signals could be detected on the dentrites of DIV7 and DIV14 neurons as indicated in the NG group of Fig. 2A. However, synapsin I positive (Syn^+^) signals were mainly distributed in the cell bodies on the DIV7 cortical neurons, but Syn^+^ signals could be detected on the dentrites and cell bodies of the DIV14 cortical neurons in NG group, which phenomenon was consistent with previous report (Chai et al. 2016). We Further detected that high glucose treatment significantly reduced the fluorescent signals of PSD95^+^ and Syn^+^ puncta as indicated in the HG group of Fig. 2A and B compared with that of the NG group. To verify the effect of high glucose on synapse formation, we further used western blot analyse to semi-quantify the levels of PSD95 and Synapsin I protein expression. The results showed that high glucose treatment significantly decreased the expressive levels of PSD95 and Synapsin I protein in DIV7 neurons (*p*_PSD95_ = 0.026; *p*_Synapsin I_ = 0.001; Fig. 2C) and DIV14 neurons (*p*_PSD95_ =0.015; *p*_Synapsin I_ =0.033; Fig. 2D) compared to normal glucose treatment. These results suggest that hyperglycaemic stimulation can reduce the expression of postsynaptic and presynaptic proteins in the culture neurons, thus causing the dysfunction of synaptic development.

### BMECs Rescue High Glucose-Induced Neuronal Morphology Aberrations

To determine the effects of vascular endothelial cells on HG-induced neuronal morphology aberrations, DIV7 and DIV14 neurons were cocultured with BMECs for 24 h (Fig. 1A), and Map2 immunofluorescence staining was performed to reveal the neuritic outgrowth (Fig. 1B). As shown in Fig. 1C-a and D-a, the average process lengths (µm) of DIV7 and DIV14 neurons cocultured with BMECs under high-glucose condition (the HG-BMECs group) were 299.65 (235.13, 337.89) and 420.33 (311.61, 549.02), respectively, which were significantly increased compared with that in the HG group (*p*_DIV7_ < 0.0001; *p*_DIV14_< 0.0001; Table 1). Moreover, as shown in Fig. 1C-b, the numbers of process intersections at 20, 30, 40 and 50 μm from the cell body were significantly increased in DIV7 neurons cocultured with BMECs (the HG + BMEC group) compared with that in the neuron culture alone (the HG group) (*p*_20µm_ = 0.001; *p*_30µm_ < 0.0001; *p*_40µm_ < 0.0001; *p*_50µm_ < 0.0001; Supplementary Material). As shown in Fig. 1D-b, the numbers of process intersections of DIV14 neurons in HG + BMECs group were also significantly increase at 20, 30, 40, 50, 60 ,70, 80 and 90 μm from the cell body compared to that in the HG group (*p*_20µm_ =0.003; *p*_30µm_ < 0.001;*p*_40µm_ < 0.0001; *p*_50µm_ < 0.001; *p*_60µm_ < 0.001; *p*_70µm_ < 0.001; *p*_80µm_ =0.001; *p*_90µm_ =0.001; Supplementary Material). To further compare the influence of BMECs treatment on the morphological complexity of DIV7 and DIV14 neurons under high-glucose conditions, the average numbers of process intersections were statistically analysed. As shown in Fig. 1C-c and D-c, the average numbers of process intersections of DIV7 and DIV14 neurons in the HG + BMECs group were 28.00 (21.00, 32.50) and 41.00 (27.50, 49.50), respectively, which were significantly higher than that in the HG group (*p*_DIV7_ < 0.0001; *p*_DIV14_ < 0.0001; Table 1). These results suggest that endothelial cells can ameliorate neuronal damage induced by high glucose stimulation.

To further explore whether endothelial cells exert this biological effect by secreting certain nutritional factors, neurons were cultured with B-CM under high glucose condition (in the HG + B-CM group). As shown in Fig. 1C-a and D-a, the average process lengths (µm) of DIV7 and DIV14 neurons in the HG + B-CM group were 354.57 (328.24, 422.81) and 522.26 (407.08, 633.31), respectively, which were significantly longer compared with that in the HG group (*p*_DIV7_ <0.0001; *p*_DIV14_ < 0.0001; Table 1). Moreover, as shown in Fig. 1C-b, the numbers of process intersections at 20 to 90 μm from the cell body in the HG + B-CM group were significantly increased for DIV7 neurons (*p*_20µm_ < 0.0001; *p*_30µm_ < 0.0001; *p*_40µm_ < 0.0001; *p*_50µm_ < 0.0001; Supplementary Material) and DIV14 neurons (*p*_20µm_ = 0.002; *p*_30µm_ <0.0001; *p*_40µm_ < 0.0001; *p*_50µm_ < 0.0001; *p*_60µm_ < 0.0001; *p*_70µm_ < 0.0001; *p*_80µm_ < 0.0001; *p*_90µm_ < 0.001; Supplementary Material) compared to that in the HG group. Figure 1C-c and D-c showed that the average numbers of process intersections of DIV7 and DIV14 neurons in the HG + B-CM group were 34.00 (29.00, 38.50) and 42.00 (38.50, 59.50), respectively, indicating that B-CM treatment could significantly alleviate neuronal process damage induced by high glucose treatment (*p*_DIV7_ < 0.0001; *p*_DIV14_ < 0.0001; Table 1). As shown in Fig. 2, fluorescent signals of PSD95^+^ and Syn ^+^ puncta and their protein expression in the neurons were higher in the HG + B-CM group than that in the HG group. Statistical analysis data showed that PSD95 and Synapsin I protein expression were significantly upregulated in the HG + BMEC and in the HG + B-CM group compared with that in the HG group DIV7 neurons (*p*_BMEC−PSD95_= 0.049; *p*_BMEC−Synapsin I_ = 0.011; *p*_B−CM−PSD95_= 0.023; *p*_B−CM−Synapsin I_ = 0.007; Fig. 2C) and DIV14 neurons (*p*_BMEC−PSD95_= 0.024; *p*_BMEC−Synapsin I_ = 0.023; *p*_B−CM−PSD95_= 0.015; *p*_B−CM−Synapsin I_= 0.023; Fig. 2D). These results suggest that endothelial cells protect against the HG-induced reduction of synapse formation.

### BMECs Exert Neuroprotective Effects Against High Glucose-Induced Neuronal Morphology Aberrations Through Activation of VEGF Receptors

In cerebral ischaemia models, VEGF inhibits hypoxic death of cortical neurons and promotes neurogenesis through neuroprotection and neural regeneration (Jin et al. 2002; Rosell et al. 2013; Shen et al. 2016; Wang et al. 2009; Wu et al. 2019). Therefore, we explored whether endothelial cell-conditioned medium protects against the HG-induced reduction in neuronal growth and synaptic development through VEGF. Neurons were pre-treated with SU1498 (SU), a VEGF receptor inhibitor, at final concentration of 10 µM for 1 h and then incubated with normal glucose culture medium (NG + SU) or HG + B-CM medium (HG + B-CM + SU) for 24 h (Fig. 1A). Map2 immunofluorescence staining was performed after treatment (Fig. 1B). The length and complexity of neurites were analysed by Neuron J plug-in of ImageJ software and the Sholl Analysis plug-in as mentioned above. As shown in Fig. 1C-a and D-a, the average process lengths (µm) of DIV7 and DIV14 neurons in the NG + SU group were no different from that in the NG group, indicating VEGF receptor antagonist SU1498 itself have no effect the neurite process. However, SU1498 significantly reduced the protective effects of B-CM on the HG-induced delayed of neurite process; the average process lengths (µm) of DIV7 and DIV14 neurons in the HG + B-CM + SU group were 139.46 (96.75, 181.09) and 191.03 (156.47, 273.86), respectively, which were significantly lower than 354.57 (328.24, 422.81) and 522.26 (407.08, 633.31) in the HG + B-CM group (*p*_DIV7_ <0.0001; *p*_DIV14_ <0.0001; Table 1).

Moreover, as shown in Fig. 1C-b and D-b, the total numbers of process intersections at different distance from the cell body were significantly reduced on DIV7 neurons (*p*_20µm_ =0.002; *p*_30µm_ <0.0001; *p*_40µm_ <0.0001; *p*_50µm_ <0.0001; Supplementary Material) and DIV 14 neurons (*p*_20µm_ = 0.005; *p*_30µm_ <0.0001; *p*_40µm_ <0.0001; *p*_50µm_ <0.0001; *p*_60µm_ <0.0001; *p*_70µm_ <0.0001; *p*_80µm_ <0.0001; *p*_90µm_ <0.001; Supplementary Material) in the HG + B-CM + SU group compared with that in the HG + B-CM group, respectively. The statistical data showed that the average numbers of process intersections for DIV7 and DIV14 neurons were 13.00 (10.00, 16.00) (*p* < 0.0001; Table 1) and 19.00 (15.50, 26.00) (*p* < 0.0001; Table 1), respectively, in the HG + B-CM + SU group, which were significantly lower than that the in the HG + B-CM group (Fig. 1C-c and D-c). These results indicate that blocking the VEGF receptor can inhibit the protective effect of B-CM on HG-induced neuronal process injury while itself did not show any effect on normal neurite process (Fig. 1).

With the same model, we further analyzed effects of SU1498 on the synaptic development of cultured neurons in the HG + B-CM treatment. The fluorescent signals of PSD95^+^ and Syn^+^ puncta (Fig. 2A and B) and their protein expression (Fig. 2C and D) in DIV7 neurons (*p*_PSD95_ =0.049; *p*_Synapsin I_ =0.020; Fig. 2C) and DIV14 neurons (*p*_PSD95_ =0.029; *p*_Synapsin I_ =0.026; Fig. 2D) were significantly decreased in the HG + B-CM + SU1498 group compared with that in the HG + B-CM group. Furthermore, SU1498 treatment (the NG + SU group) did not change synaptic formation on DIV7 and DIV14 neurons in the NG group. The results suggest that BMECs coculture produce neuroprotection against HG-induced the dysfunction of neurite outgrowth and synaptic development through the activation of VEGFR-2.

**Table 1:** Dendrite morphology of cortical neurons of Fig. 1

DIV7 (*n* = 25)^a^	Total dendritic length (µm)	Total intersections points (no.)
NG	247.21 (210.45, 289.65)	22.00 (19.00, 26.50)
HG	117.18 (89.22, 133.21)****	11.00 (9.00, 13.00)****
HG + BMEC	299.65 (235.13, 337.89)^####^	28.00 (21.00, 32.50) ^####^
HG + B-CM	354.57 (328.24, 422.81)^####^	34.00 (29.00, 38.50) ^####^
HG + B-CM + SU	139.46 (96.75, 181.09)^&&&&^	13.00 (10.00, 16.00) ^&&&&^
NG + SU	253.44 (182.51, 304.16)	22.00 (17.00, 26.00)
*DIV14 (n = 25)*^a^		
NG	396.76 (347.98, 473.43)	38.00 (31.50, 43.00)
HG	198.84 (143.43, 245.98)****	17.00 (14.00, 22.50)****
HG + BMEC	420.33 (311.61, 549.02) ^####^	41.00 (27.50, 49.50) ^####^
HG + B-CM	522.26 (407.08, 633.31) ^####^	42.00 (38.50, 59.50) ^####^
HG + B-CM + SU	191.03 (156.47, 273.86) ^&&&&^	19.00 (15.50, 26.00) ^&&&&^
NG + SU	345.88 (287.29, 411.85)	32.00 (26.50, 40.50)

NG, neurons cultured alone and treated with 25 mM glucose; HG, neurons cultured alone and treated with 45 mM glucose; HG+BMEC, neurons grown with BMECs in 45 mM glucose; HG+B−CM, neurons treated with B−CM and 45 mM glucose; HG+B−CM+ SU, neurons treated with B−CM, 45 mM glucose and 10 µM SU1498; NG+SU, neurons cultured with 10 µM SU1498 and treated with 25 mM glucose

****p < 0.0001 vs. NG; ####*p* <0.0001 vs. HG; &&&&*p* <0.0001 vs. HG +B−CM

^a^Median and interquartile range

#### VEGF Ameliorates High Glucose-Induced Neuronal Morphology Aberrations

**Fig. 3:**
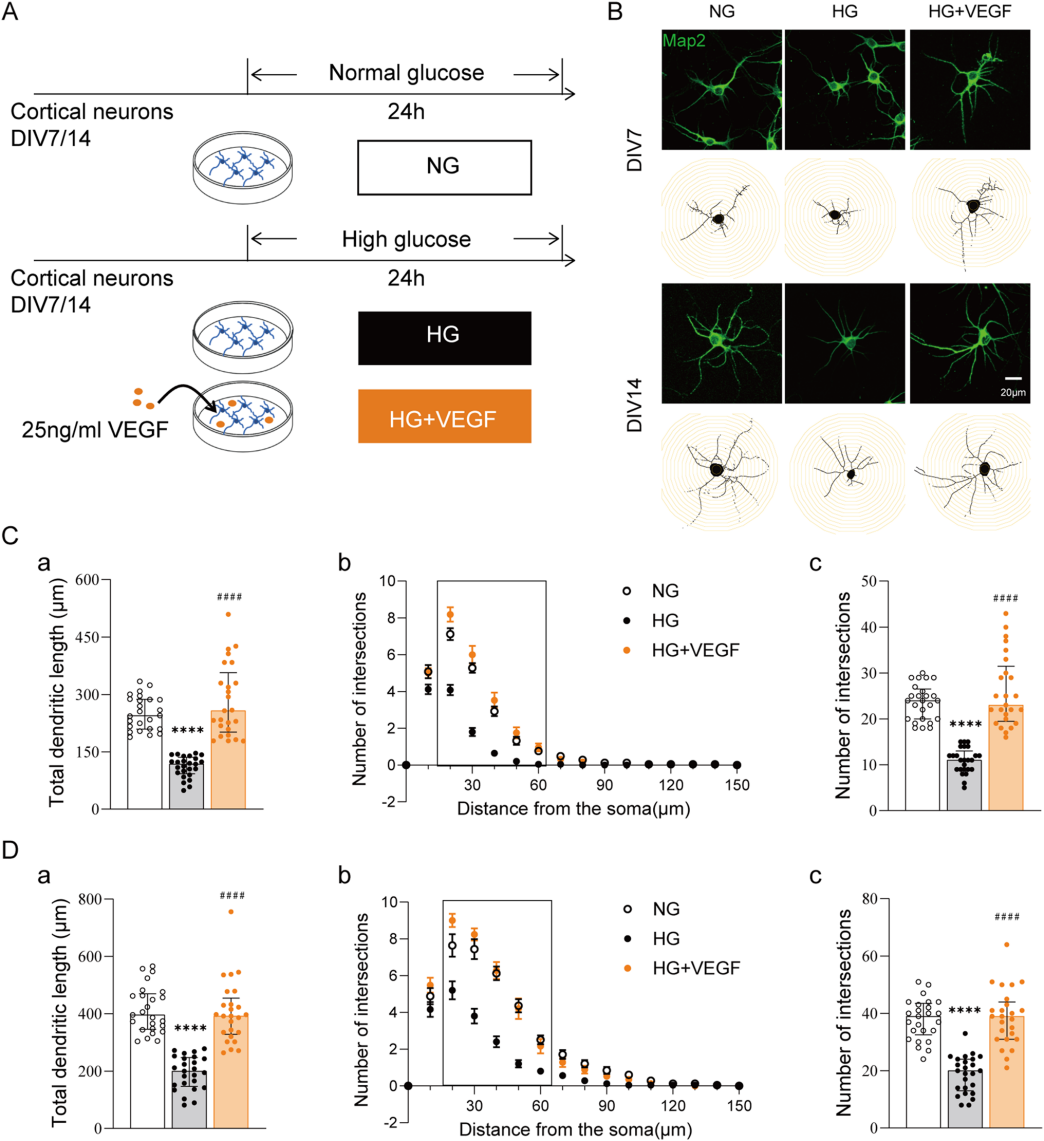
VEGF rescues the high glucose−induced reduction in dendritic outgrowth of cortical neurons. **A** Schematic of the experimental procedures. **B** Confocal images of staining for the somatodendritic marker Map2 showing dendritic arborization and Sholl analysis of control neurons and neurons treated with high glucose without or with VEGF for 24 h. **C**–**D** Morphometric analysis of **a** the total length of dendrites and **b**−**c** Sholl analysis of dendrites of cortical neurons. **b** Plots of the numbers of intersections as a function of distance from the soma. **c** Sholl analysis was further used to quantify the total numbers of process intersections. *n* = 25. ****p <0.0001 vs. NG; ####*p* <0.0001 vs. HG. Bars: 20 μm. The data are presented as the median and interquartile range or mean ± SD. NG, neurons cultured alone and treated with 25 mM glucose; HG, neurons cultured alone and treated with 45 mM glucose; HG + VEGF, neurons treated with 45 mM glucose and 25 ng/ml VEGF

In order to further investigate whether VEGF can exert the same protective effect as endothelial cells on neurons under high-glucose conditions, we exogenously administrated VEGF at final concentration of 25 ng/mL into DIV7 and DIV14 neurons for 24 h under high glucose condition (Fig. 3A). Map2 immunofluorescence staining was performed to reveal the neurites (Fig. 3B). The Neuron J plug-in of ImageJ software and the Sholl Analysis plug-in were used to analyse process length and complexity (Fig. 3C–D). As shown in Fig. 3C-a and D-a, the neurons in the HG + VEGF group showed that the average process lengths (µm) of DIV7 and DIV14 neurons were 257.51 (201.20, 356.89) and 391.63 (328.07, 454.82), respectively, which were significantly longer compared with that in the HG group (*p*_DIV7_ <0.0001; *p*_DIV14_ <0.0001; Table 2).

Figure 3 C-b and D-b showed that total numbers of process intersections at 20, 30, 40, 50 and 60 μm from the cell body were significantly increased for DIV7 and DIV14 neurons in the HG + VEGF group compared with that in the HG group (*p*_DIV7−20 μm_ <0.0001; *p*_DIV7−30 μm_ <0.0001; *p*_DIV7−40 μm_ <0.0001; *p*_DIV7−50 μm_ <0.0001; *p*_DIV7−60 μm_ <0.001; *p*_DIV14−20 μm_ <0.0001; *p*_DIV14−30 μm_ <0.0001; *p*_DIV14−40 μm_ <0.0001; *p*_DIV14−50 μm_ <0.0001; *p*_DIV14−60 μm_ = 0.006; Supplementary Material). The average numbers of process intersections for DIV7 and DIV14 neurons in the HG + VEGF group were 23.00 (19.50, 31.50) and 39.00 (31.00, 44.00), respectively, which were significantly higher than that in the HG group (*p*_DIV7_ < 0.0001; *p*_DIV14_ < 0.0001; Table 2). The above experimental results indicate that exogenous addition of VEGF protein protects against neuronal process injury induced by high glucose.

**Fig. 4:**
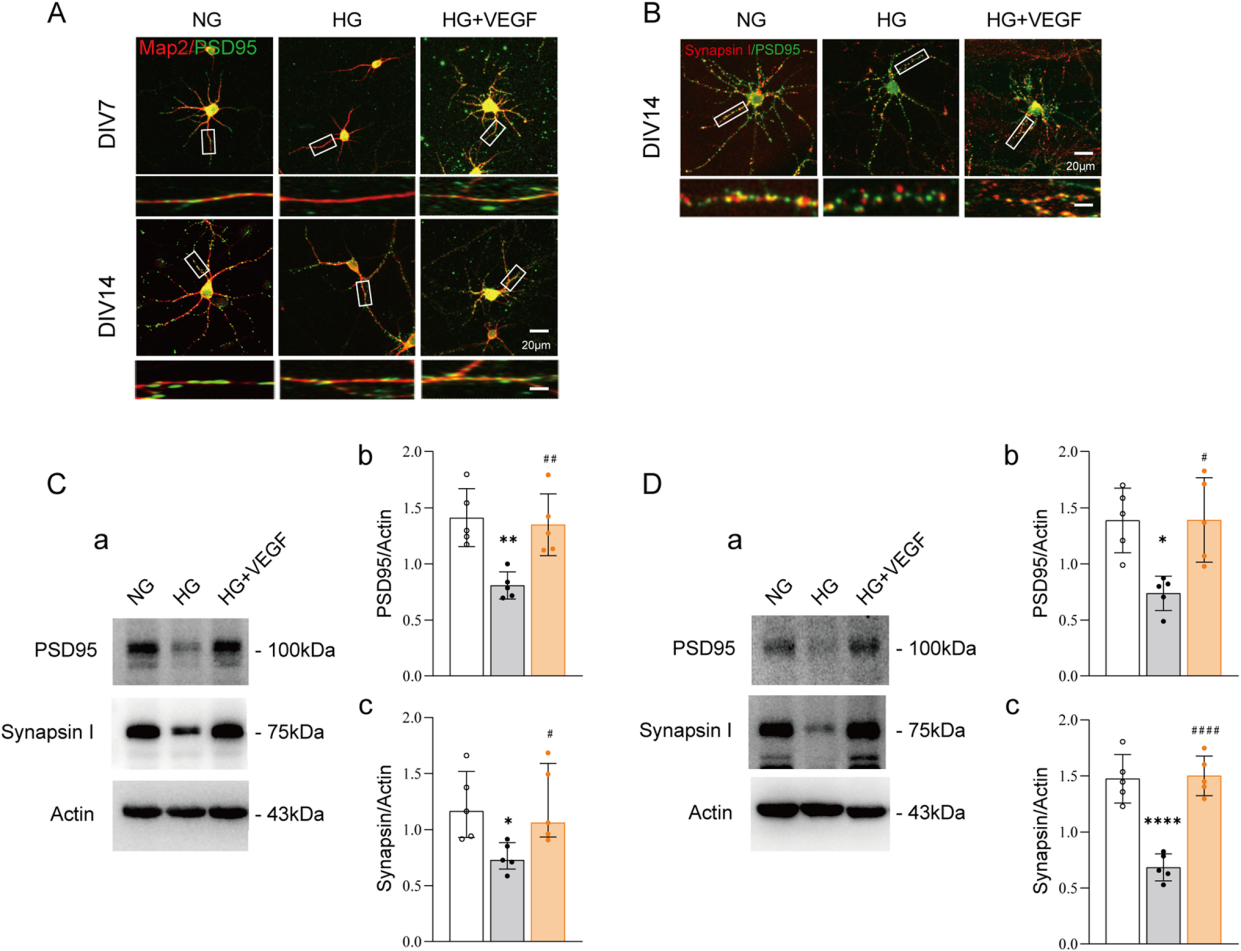
VEGF rescues the high glucose−induced reduction in synaptogenesis of cortical neurons. **A**, **B** Representative images of immunostaining of PSD95 (green) and the dendritic marker protein Map2 (red) or PSD95 (green) and the general presynaptic marker protein Synapsin I (red) in neurons treated with high glucose without or with VEGF for 24 h. **C**, **D** Immunoblots of PSD95 and Synapsin I in cultured cortical neurons. Actin was used for normalization. *n* = 5. *p < 0.05, **p < 0.01, ****p <0.0001 vs. NG; #*p* <0.05, ##*p* < 0.01, ####*p* <0.0001 vs. HG. Bars: 20 μm; magnification: 5 μm. The data are presented as the median and interquartile range or mean ± SD. NG, neurons cultured alone and treated with 25 mM glucose; HG, neurons cultured alone and treated with 45 mM glucose; HG + VEGF, neurons treated with 45 mM glucose and 25 ng/ml VEGF

Further study found that exogenous addition of VEGF protein to DIV7 and DIV14 neurons significantly increased the density of PSD95^+^ and Syn^+^ puncta as well as the levels of PSD95 and Synapsin I expression in DIV7 and DIV14 neurons in the HG + VEGF group compared with that in the HG group (*p*_DIV7−PSD95_=0.006; *p*_DIV7−Synapsin I_ =0.033; *p*_DIV14−PSD95_ = 0.011; *p*_DIV14−Synapsin I_ <0.0001; Fig. 4C–D). These results indicate that VEGF exerts the same protective effect as BMECs against HG-induced damage/delayed neuronal synapsis formation.

**Table 2:** Dendrite morphology of cortical neurons of Fig. 3

DIV7 (*n* = 25)^a^	Total dendritic length (µm)	Total intersections points (no.)
NG	244.91(209.67, 287.38)	24.00 (20.00, 26.50)
HG	117.99(92.59, 135.50)****	11.00 (9.00, 13.00) ^****^
HG + VEGF	257.51(201.20, 356.89) ^####^	23.00 (19.50, 31.50) ^####^
*DIV14 (n = 25)*^a^		
NG	396.11(346.03, 469.54)	39.00 (32.50, 43.50)
HG	200.05(146.49,246.73)****	20.00 (13.00, 24.00)****
HG + VEGF	391.63(328.07, 454.82) ^####^	39.00 (31.00, 44.00) ^####^

****p <0.0001 vs. NG; ####*p* <0.0001 vs. HG.NG, neurons cultured alone and treated with 25 mM glucose; HG, neurons cultured alone and treated with 45 mM glucose; HG+VEGF, neurons treated with 45 mM glucose and 25 ng/ml VEGF

^a^Median and interquartile range

### BMECs Rescue the High Glucose-Induced Reduction of in 2-NBDG Uptake by Cortical Neurons Through Activation of VEGF Receptors

**Fig. 5:**
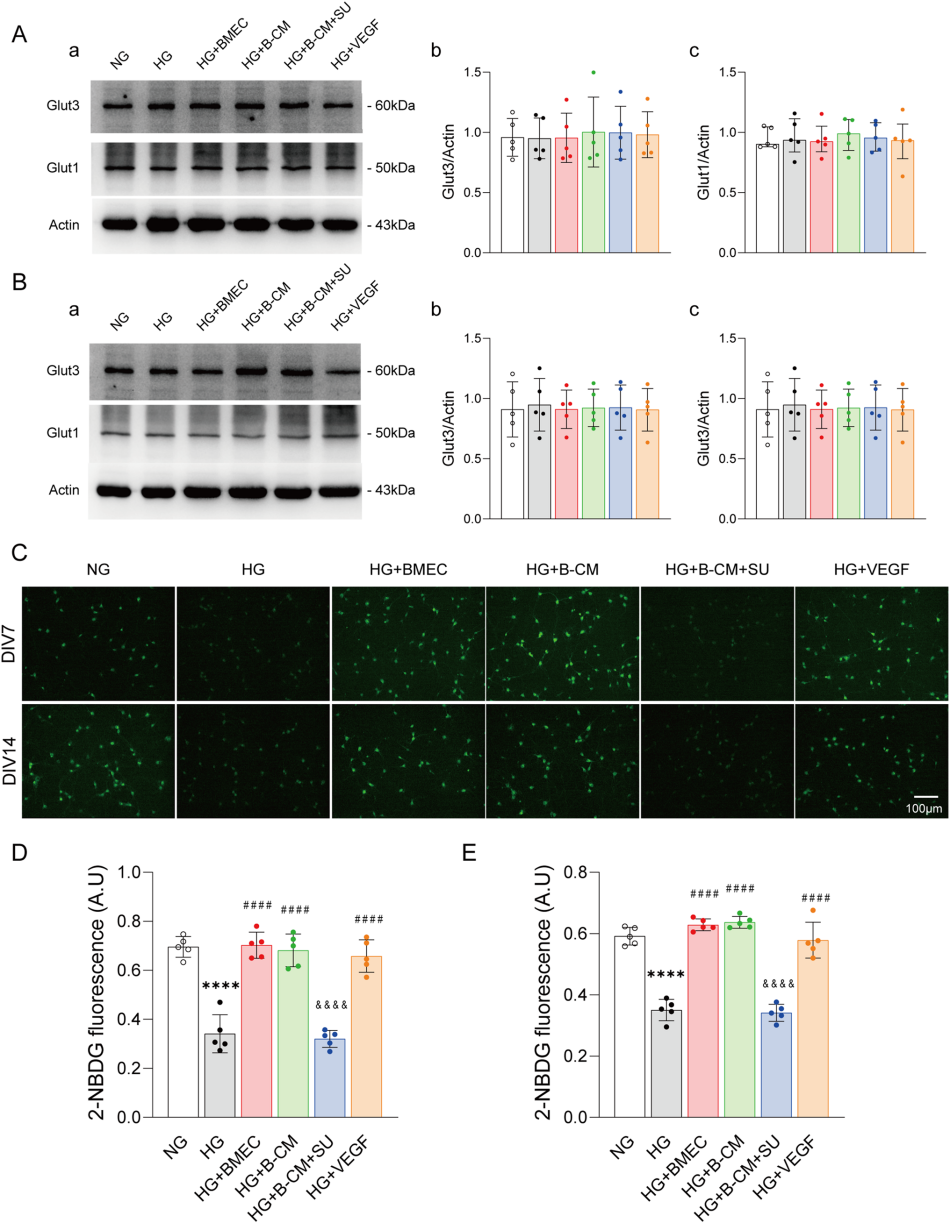
BMECs ameliorate the high glucose−induced reduction in 2−NBDG uptake by cortical neurons through activation of VEGF receptors. **A**−**B** Representative western blot of Glut3 and Glut1 in DIV7 and DIV14 cortical neurons. **C** Twenty−four−hour 2−NBDG accumulation in cortical neurons treated with BMEC, B−CM or B−CM and SU1498 (10 µM) and VEGF under high−glucose conditions for 24 h. **D**−**E** Quantitative analysis of 2−NBDG accumulation in Fig. 5C. *n* =5. ****p < 0.0001 vs. NG; ####*p* <0.0001 vs. HG; &&&&*p* <0.0001 vs. HG +B−CM. Scale bar = 100 μm. The data are presented as the median and interquartile range or mean ± SD. NG, neurons cultured alone and treated with 25 mM glucose; HG, neurons cultured alone and treated with 45 mM glucose; HG+BMEC, neurons grown with BMECs in 45 mM glucose; HG+B−CM, neurons treated with B−CM and 45 mM glucose; HG+B−CM+ SU, neurons treated with B−CM, 45 mM glucose and 10 µM SU1498; HG+VEGF, neurons treated with 45 mM glucose and 25 ng/ml VEGF

It has been reported that obesity can inhibit Glut1 levels and glucose metabolism in the brain (Jais et al. 2016). In this study, we further investigated the effects of different treatment, as mentioned above, on the protein level and glucose uptake function of glucose transporters in DIV7 and DIV14 neurons under high glucose treated condition. We observed that there was no significant difference in the levels of Glut3 and Glut1 proteins expression among all group as shown in Fig. 5A–B. The results indicated those treatment did not change the protein expression levels. Then, 2-NBDG living cell image was used to analyze the function of glucose uptake of neuronal glucose transporters. For this study, neurons were incubated with 50 µM 2-NBDG for 24 h and immunofluorescence signals in the neurons were captured under a confocal microscope at a wavelength of 488 nm every 2 s for 5 min. Ten neurons were randomly selected from each group to calculate the average fluorescence intensity. According to this method, the greater the intensity of the neuron’s fluorescence, the more glucose the neuron is taking in. The results showed that HG treatment significantly reduced fluorescent intensity in DIV7 and DIV14 neurons compared with NG treatment (As shown in Fig. 5C). More interestingly, BMECs, B-CM or VEGF treatment respectively prevented HG-induced inhibition of glucose uptake in DIV7 and DIV14 neurons. The quantitative data showed that intensity of 2-NBDG fluorescence was significantly enhanced in DIV7 and DIV14 neurons in the HG + BMECs (*p*_DIV7_ <0.0001; *p*_DIV14_ <0.0001), HG + B-CM (*p*_DIV7_ <0.0001; *p*_DIV14_ <0.0001) and HG + VEGF (*p*_DIV7_ <0.0001; *p*_DIV14_ <0.0001) group compared with that in the HG group, respectively, as shown in Fig. 5D–E. Moreover, we also found that restoration of B-CM treatment in the HG-induced inhibition of glucose uptake could be antagonized by SU1498, a VEGF receptor antagonist (Fig. 5D–E). These results suggest that the BMECs and B-CM restore glucose uptake capacity under hyperglycaemia condition via the activation of VEGF receptors through the release of VEGF from endothelial cells.

## Discussion

In this study, we used coculture of BMECs and cortical neurons and combined with a hyperglycaemic model to reveal that cerebral microvascular endothelial cells protected neurons against HG-induced delayed neurite outgrowth and synaptogenesis as well as inefficient glucose uptake through the activation of VEGF receptors via VEGF release from endothelial cells. These results also contribute to our understanding of the cellular and molecular mechanisms underlying neurodegeneration in diabetic brain.

As the basic unit of neural networks, neurons play a crucial role in higher brain functions such as learning and memory. Dendrites, as projections that branch out from the cell bodies of neurons, account for approximately 95% of the total volume of neurons; thus, quantitative analysis of dendrites can reveal subtle changes in dendritic morphology and neuroplasticity (Fiala et al. 2002). The complexity of dendritic structure determines the biological characteristics of dendrites to a great extent and thus affects their function (van Elburg and van Ooyen 2010). This study showed that acute hyperglycaemia reduced the length and complexity of neuronal dendrites (Fig. 1). Our results directly explain previous results reported by Dr. Yan’s laboratory (Yan et al. 2016); they found reduction of dendritic branch numbers and spine density in the parietal cortex and impairment of learning and memory in diabetic rats. Thus, both in vitro and in vivo studies indicate that hyperglycaemia reduces integration of neurons into neural circuits, leading to neurotransmission deficits and brain dysfunction. We also found that high glucose treatment decreased the expression of presynaptic (Synapsin I) and postsynaptic (PSD95) proteins (Fig. 2). PSD95 is a key element for maintaining synaptic function and synaptic connections (El-Husseini et al. 2000). The loss of synaptic vesicle proteins and PSD95 directly causes to degeneration of dendritic spines and impairment of recognition and spatial memory (Schmitt et al. 2009; Shao et al. 2011). Therefore, reduced neurite outgrowth and PSD95 expression may impair the function of dendritic spines and even disrupt synaptic connections in the cerebral cortex, thereby leading to cognitive decline and the development of neurodegenerative diseases such as diabetic dementia.

Functional neurovascular interactions in the brain underlie the maintenance of brain function. It has been proposed that neurodegeneration in diabetic brain might be caused by dysfunction of the NVU (Yan et al. 2020; Zhao et al. 2013). In this experiment, the neurons cocultured with endothelial cells had less neurite damage induced by hyperglycaemia than neurons cultured alone. Furthermore, treatment with endothelial cell-conditioned medium had similar effects as coculture with endothelial cells on the HG-treated neurons, as it could alleviate the pathological changes in neurons induced by acute high glucose stimulation (Figs. 1 and 2). Combined with previous results that in vivo engraftment of endothelial cells increased synaptic puncta and excitatory postsynaptic currents in layers 2/3 of the motor cortex and were capable of enhancing angiogenesis and synaptogenesis and improved motor function in ischemic injury model (Wu et al. 2019), present results suggest that endothelial cells possess beneficial effects for neurites development and synaptogenesis or protect neurons against HG-induced damage.

The vascular system in the central nervous system provides nutrients and oxygen to nerve cells and regulates the function and survival of neurons and glial cells by providing neurotrophic factors (Echeverria et al. 2017). VEGF, a secreting factor from vascular endothelial cells, plays a crucial role in the development of the nervous system by regulating neurogenesis, neuronal development and the differentiation and formation of cerebral vessels (Kirby et al. 2015; Lei et al. 2023; Sun and Guo 2005). In our previous study, we reported that BMECs conditioned medium enhanced spine and synapse formation in culture neurons and protected against hypoxic/ischemic neuronal injury. However, after removal of VEGF in conditioned medium with VEGF antibody or inhibition of VEGF expression in endothelial cells with VEGF shRNA, coculture with such conditional BMECs or B-CM no longer showed these effects (Wu et al. 2019). Those results clearly indicated that BMECs produce neuroprotection against hypoxic/ischemic neuronal injury and improve neurites and synaptogenesis via the release of endothelial VEGF. In this study, the pharmacological data showed that SU1498, a VEGFR-2 antagonist, blocked protective effects of BMECs and B-CM against HG-induced delayed neurite outgrowth and synaptic formation (Figs. 1 and 2), and exogenous addition of VEGF protein showed similar effects of BMECs and B-CM on HG-treated neurons (Figs. 3 and 4). However, a limitation of this study is the effect of high glucose treatment on endothelial cell VEGF release remains unclear. But we note that different results have been reported in past studies. One of these studies reported that plasma levels of VEGF were higher in newly diagnosed T2D patients than that in healthy individuals, indirectly demonstrating that hyperglycemia may stimulate VEGF release in endothelial cells in the early stages of diabetes(Sun et al. 2019). This results indirectly suggest that high-glucose treatment in this experiment might cause release VEGF from the BMECs. Putting all together, we speculate that vascular endothelial cells attenuate HG-induced neuronal injury or deficits of dendritic development and synaptogenesis via the activation of VEGF receptors and release of VEGF from the endothelial cells.

Peripheral glucose entry into neurons mainly depends on Glut1 in the BBB and Glut3 in neurons. Chronic hyperglycaemia downregulate the glucose transporter in the BBB (Hasselbalch et al. 2001). In present study, we found decrease of glucose uptake capacity in cortical neurons following high glucose treatment (Fig. 5). In the clinic study, it has been reported that impaired glucose regulation and insufficient energy metabolism in the brains of patients with diabetes lead to neuronal malnutrition and cognitive dysfunction (Sickmann and Waagepetersen 2015). In diabetic animals, it has been found that the amount of choline that enters the brain through the BBB is decreased (Mooradian 1987) and acetyl-CoA activity is reduced (Sickmann et al. 2010), resulting in a decrease in acetylcholine (ACh) content in the brain. ACh is an important neurotransmitter in the brain and participates in the regulation of cognitive function and reduction of ACh levels in the brain further exacerbates cognitive impairment. These results indicate that changes in glucose levels in the brain are not only related to energy supply for the brain, but also affect the synthesis of ACh neurotransmitters. More interestingly, this study found that BMECs or B-CM could significantly alleviate HG-induced inhibition of neuronal glucose uptake in a SU1498 antagonized manner (Fig. 5), and VEGF treatment had the same effect as BMECs or B-CM (Fig. 5). This result suggest that endothelial VEGF regulates neuronal glucose uptake in the neurons following HG-stimulation. Interestingly, diabetic mice induced by high-fat diet also showed reduction of glucose uptake and Glut1 expression in the brain (Jais et al. 2016). However, the resulting inflammation leads to an increase in VEGF production in macrophages, which in turn restored Glut1 levels and glucose metabolism in the brain, thereby maintaining cognitive function (Jais et al. 2016). The same phenomenon has been observed in clinical studies (Schüler et al. 2018). Collectively, this suggests that VEGF is beneficial for maintaining glucose uptake or utilization in neurons of the brain with hyperglycaemia.

Epidemiological studies have shown that the patients with diabetes become a risk factor of neurodegenerative diseases and dementia (Schüler et al. 2018). AD, the most common type of dementia, is closely associated with T2D and impaired glucose and energy metabolism in the brain (Yan et al. 2020). A positron emission tomography (PET) study confirmed that, in the patients with mild cognitive impairment (MCI), glucose uptake in the entorhinal cortex and parietal lobe (including the precuneus) is reduced by 10–12% and decreases with the progression of AD (Cunnane et al. 2020). Patients with T2D also show abnormal glucose metabolism in the brain before occurrence of cognitive dysfunction (Barrière et al. 2018). The reduction of glucose uptake in the brain of patients with either AD or T2D before cognitive decline suggests that abnormal glucose metabolism in the brain induces and accelerates to pathogenesis of neurodegeneration in the brain at different stages of diabetic dementia. Therefore, the occurrence and progression of neurodegenerative diseases can be prevented by increasing or restoring energy metabolism in the brain. In addition, the number, size and shape of dendritic branches also altered in the AD brain, resulting in changes in synaptic structure and plasticity (Maiti et al. 2015). Many synaptic signalling proteins, such as PSD95 and proteins that regulate brain development, are also downregulated in AD brains (Gong and Lippa 2010). These signalling proteins are closely associated with dendritic spine remodelling or synaptic plasticity. Therefore, increasing brain metabolism, preserving existing synapses and reducing synaptic loss have become the main strategies for protecting cognitive function in AD. The results of this study reveal that endothelial cells can increase the uptake of glucose by neurons in the brain through VEGF, thus alleviating high glucose-induced neurite degeneration. Thus, VEGF may become a new target for the treatment of diabetes-related cognitive impairment or AD.

**Fig. 6:**
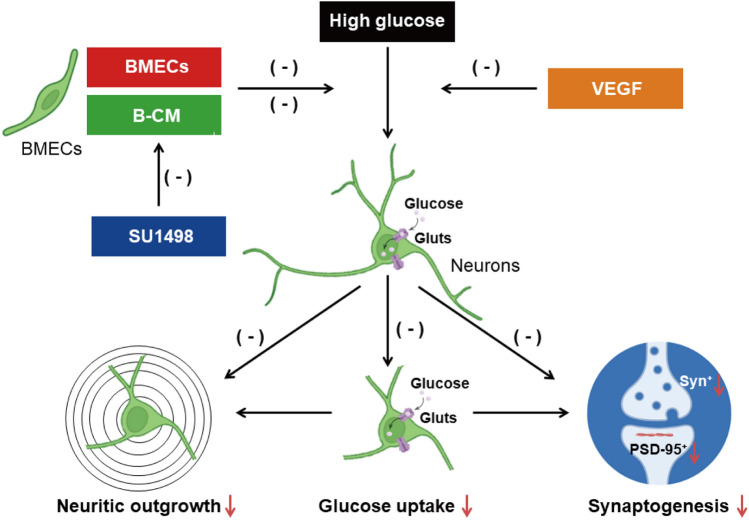
This diagram summarizes vascular endothelial cells ameliorating hyperglycemia−induced deficits in neuronal glucose uptake, neurite development, and synaptic plasticity via activation of VEGF receptors by releasing VEGF from endothelial cells. High glucose induced inhibition of neuronal glucose uptake and impaired to neuritic outgrowth and synaptogenesis. Cocultured with BMECs or B−CM, VEGF protected HG−induced inhibition of neuronal glucose uptake and impairment of neurites outgrowth and synaptogenesis, which could be antagonized by blockade of VEGF receptors. Reduction of glucose uptake may further deteriorate impairment of neurites outgrowth and synaptogenesis

In conclusion, this study revealed that endothelial cells alleviate the inhibition of neuronal glucose uptake and deficits of neurite outgrowth and synaptogenesis induced by hyperglycaemia via the activation of VEGF receptors by release of VEGF from the endothelial cells (Fig. 6). This has profound implications for the prevention and treatment of neurodegenerative diseases associated with diabetic dementia.

## Electronic Supplementary Material

Below is the link to the electronic supplementary material.

Supplementary Material 1 (PDF 2273.2 kb)

## Data Availability

The datasets generated during and/or analysed during the current study are not publicly available due to individual privacy could be compromised but are available from the corresponding author on reasonable request.
