# Supplementary material for: BMECs Ameliorate High Glucose-Induced Morphological Aberrations and Synaptic Dysfunction via VEGF-Mediated Modulation of Glucose Uptake in Cortical Neurons
Source: Cell Mol Neurobiol. 2023 Jul 7;43(7):3575–92. doi: 10.1007/s10571-023-01366-0 (PMC10477237; doi:10.1007/s10571-023-01366-0)
Supplement: Supplementary file 1 — Supplementary Material 1 (PDF 2273.2 kb) [file 10571_2023_1366_MOESM1_ESM.pdf]

BMECs ameliorate high glucose-induced morphological aberrations and synaptic dysfunction via VEGF-mediated modulation of glucose uptake by cortical neurons

Cellular and Molecular Neurobiology

Yu-Qi Huang, Xiao Gu, Xiao Chen, Yi-Ting Du, Bin-Chi Chen, Feng-Yan Sun*

*Corresponding author

Prof. Feng-Yan Sun, Department of Neurobiology, School of Basic Medical Sciences, Shanghai Medical College, Fudan University, 138 Yi-Xue-Yuan Road, Shanghai 200032, P.R. China

Tel: +86-021-54237652; Fax: +86-021-54237652. E-mail: fysun@shmu.edu.cn

Supplementary Material

I Supplementary Figures and Figure Legends

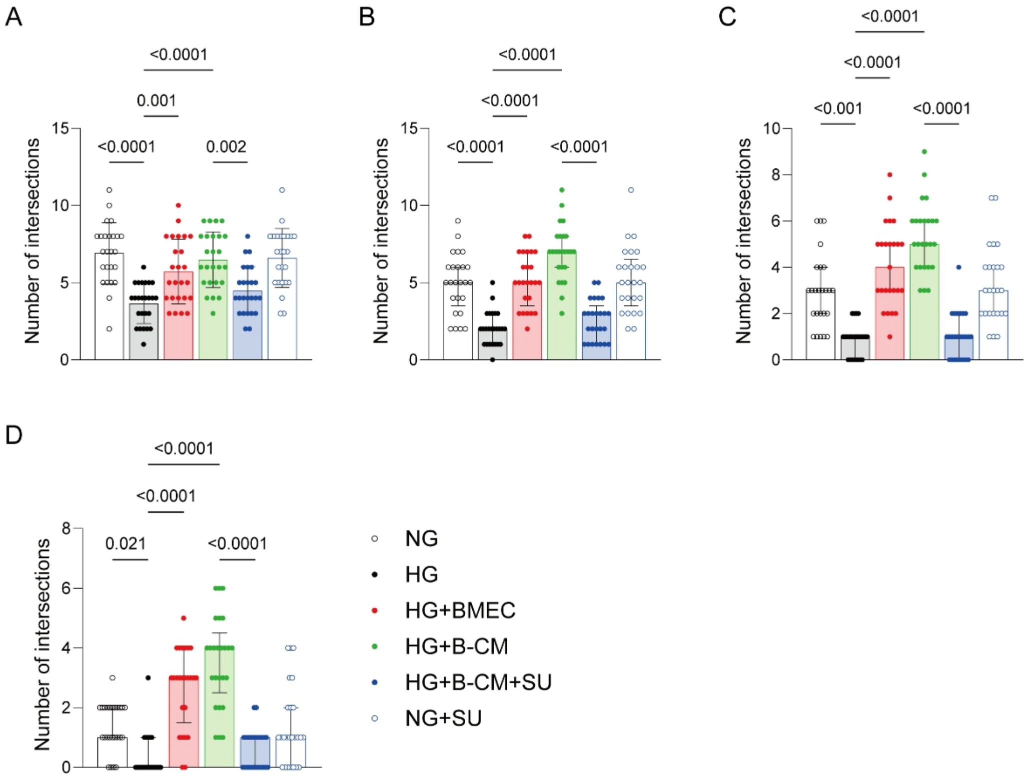

Suppl. Fig. 1C-b The number of process intersections at 20(A), 30(B), 40(C) and 50(D)μm from the cell body corresponding to the sholl analysis in Fig. 1C-b.NG, neurons cultured alone and treated with 25 mM glucose; HG, neurons cultured alone and treated with 45 mM glucose; HG+BMEC, neurons grown with BMECs in 45 mM glucose; HG+B-CM, neurons treated with B-CM and 45 mM glucose; HG+B-CM+SU, neurons treated with B-CM, 45 mM glucose and 10 μM SU1498; NG+SU, neurons cultured with 10 μM SU1498 and treated with 25 mM glucose.

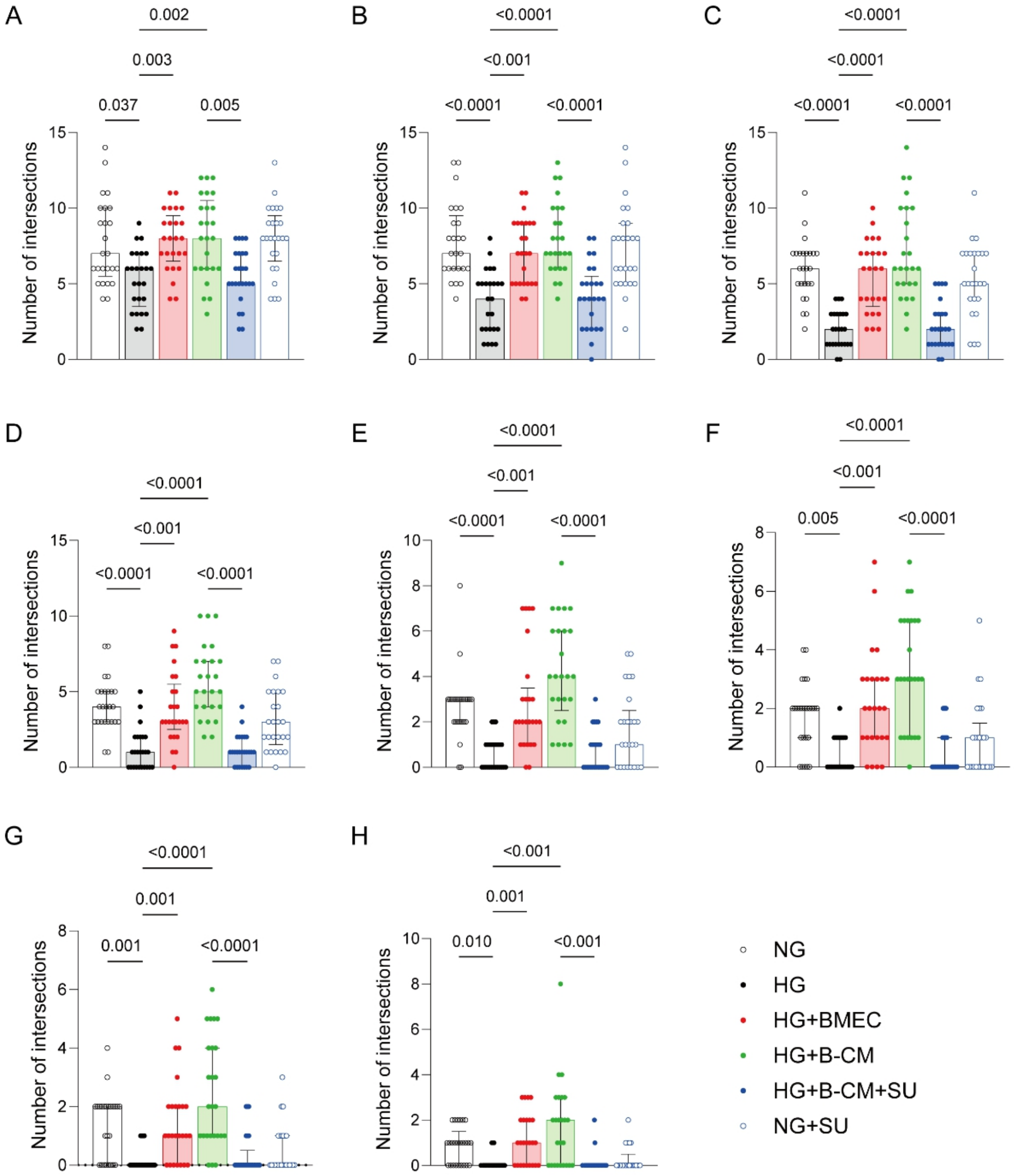

Suppl. Fig. 1D-b The number of process intersections at 20(A), 30(B), 40(C) ,50(D), 60 (E),70(F), 80(G) and 90(H)μm from the cell body corresponding to the sholl analysis in Fig. 1D-b.NG, neurons cultured alone and treated with 25 mM glucose; HG, neurons cultured alone and treated with 45 mM glucose; HG+BMEC, neurons grown with BMECs in 45 mM glucose; HG+B-CM, neurons treated with B-CM and 45 mM glucose; HG+B-CM+SU, neurons treated with B-CM, 45 mM glucose and 10 μM SU1498; NG+SU, neurons cultured with 10 μM SU1498 and treated with 25 mM glucose.

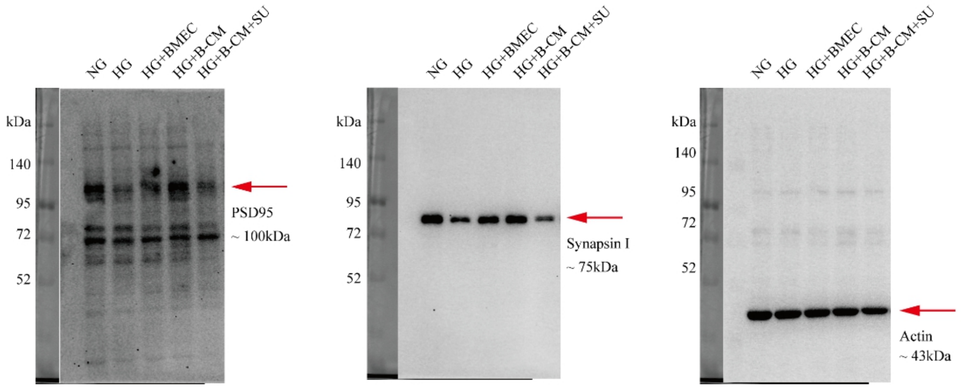

Suppl. Fig. 2C Full-size western blots corresponding to the blots shown in Fig.2C. Samples loaded on the blot correspond to: NG, neurons cultured alone and treated with 25 mM glucose; HG, neurons cultured alone and treated with 45 mM glucose; HG+BMEC, neurons grown with BMECs in 45 mM glucose; HG+B-CM, neurons treated with B-CM and 45 mM glucose; HG+B-CM+SU, neurons treated with B-CM, 45 mM glucose and 10 μM SU1498.

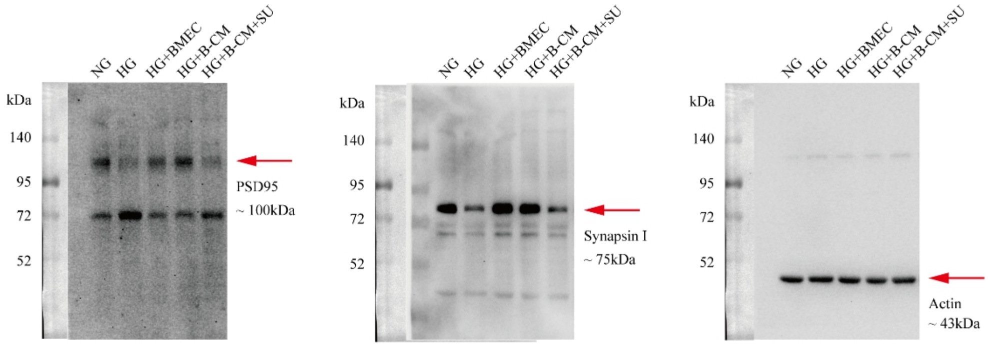

Suppl. Fig. 2D Full-size western blots corresponding to the blots shown in Fig.2D. Samples loaded on the blot correspond to: NG, neurons cultured alone and treated with 25 mM glucose; HG, neurons cultured alone and treated with 45 mM glucose; HG+BMEC, neurons grown with BMECs in 45 mM glucose; HG+B-CM, neurons treated with B-CM and 45 mM glucose; HG+B-CM+SU, neurons treated with B-CM, 45 mM glucose and 10 μM SU1498.

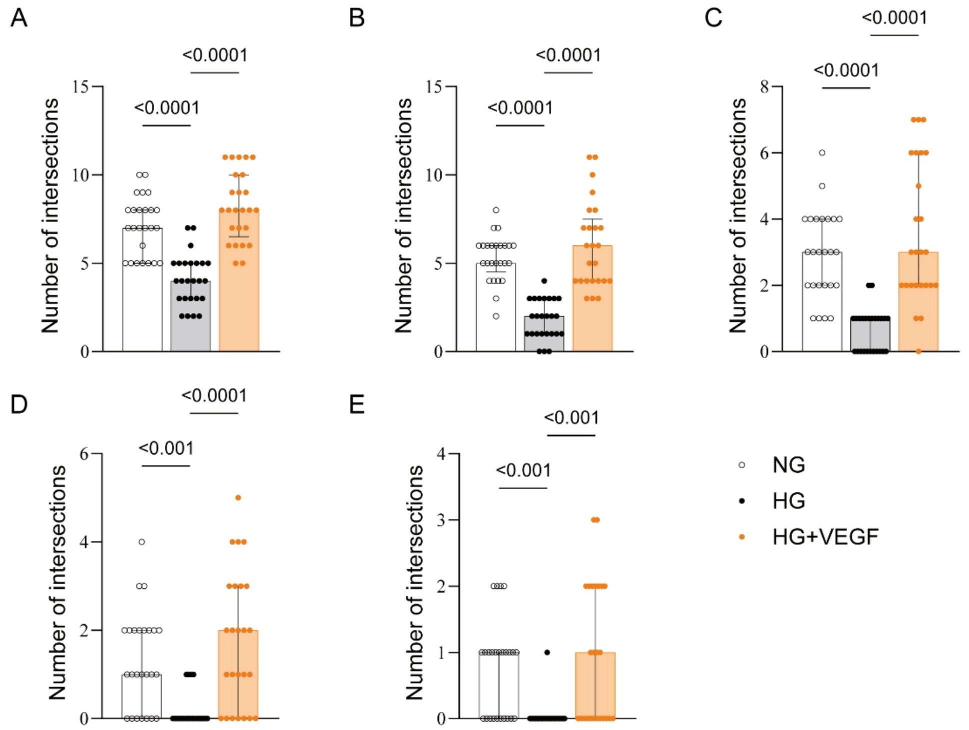

Suppl. Fig. 3C-b The number of process intersections at 20(A), 30(B), 40(C) ,50(D) and 60 (E)μm from the cell body corresponding to the sholl analysis in Fig. 3C-b.NG, neurons cultured alone and treated with 25 mM glucose; HG, neurons cultured alone and treated with 45 mM glucose; HG + VEGF, neurons treated with 45 mM glucose and 25 ng/ml VEGF.

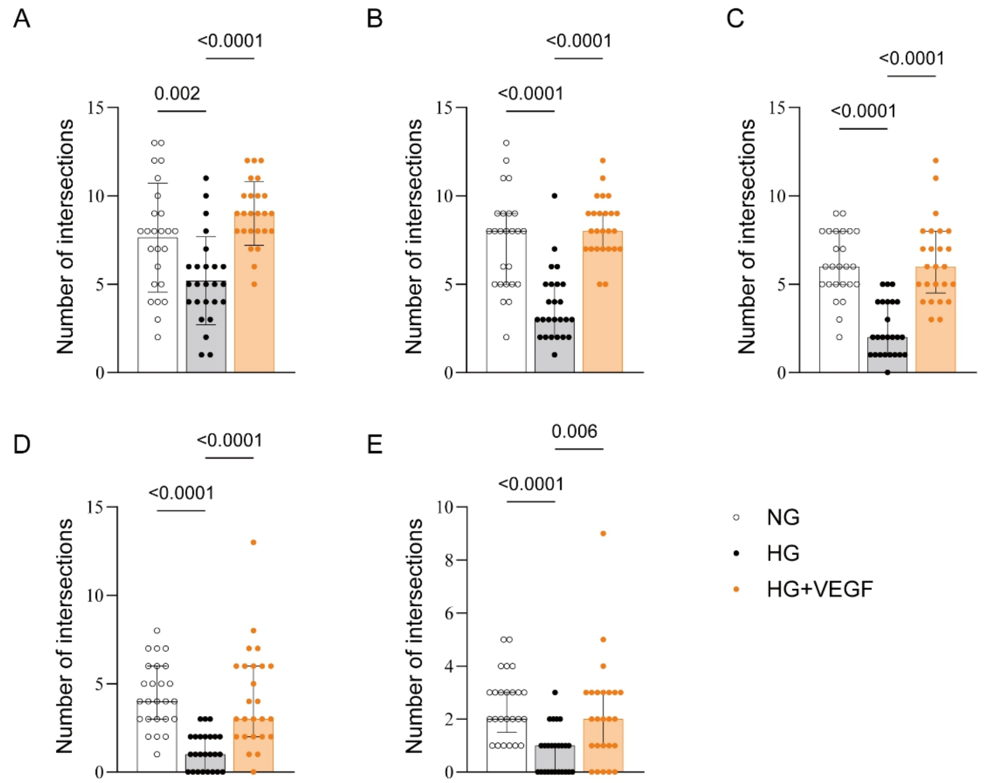

Suppl. Fig. 3D-b The number of process intersections at 20(A), 30(B), 40(C) ,50(D) and 60 (E)μm from the cell body corresponding to the sholl analysis in Fig.3 D-b.NG, neurons cultured alone and treated with 25 mM glucose; HG, neurons cultured alone and treated with 45 mM glucose; HG + VEGF, neurons treated with 45 mM glucose and 25 ng/ml VEGF.

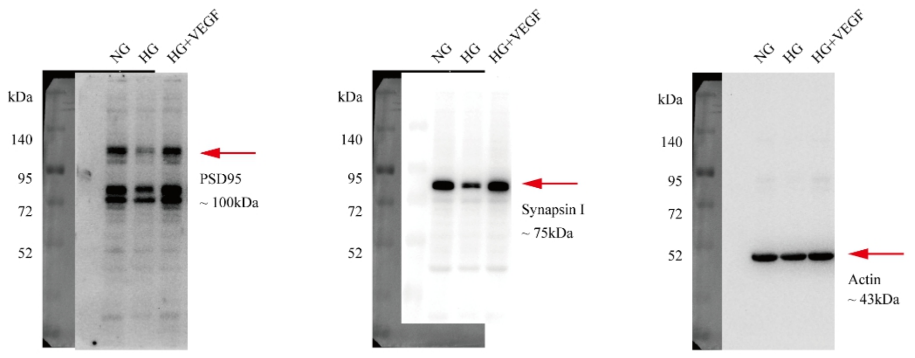

Suppl. Fig. 4C Full-size western blots corresponding to the blots shown in Fig.4C. Samples loaded on the blot correspond to: NG, neurons cultured alone and treated with 25 mM glucose; HG, neurons

cultured alone and treated with 45 mM glucose; HG + VEGF, neurons treated with 45 mM glucose and 25 ng/ml VEGF.

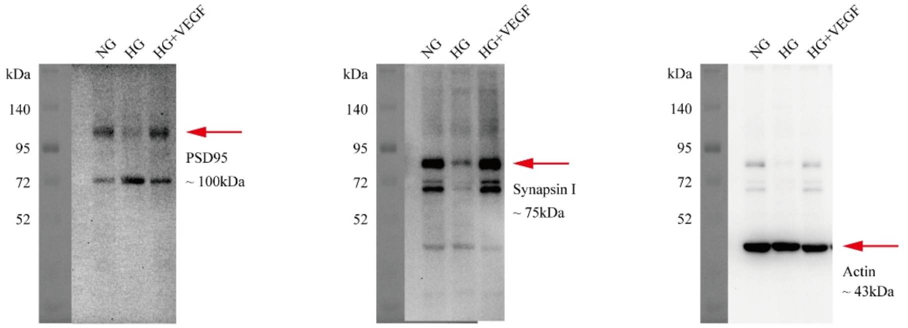

Suppl. Fig. 4D Full-size western blots corresponding to the blots shown in Fig.4D. Samples loaded on the blot correspond to: NG, neurons cultured alone and treated with 25 mM glucose; HG, neurons cultured alone and treated with 45 mM glucose; HG + VEGF, neurons treated with 45 mM glucose and 25 ng/ml VEGF.

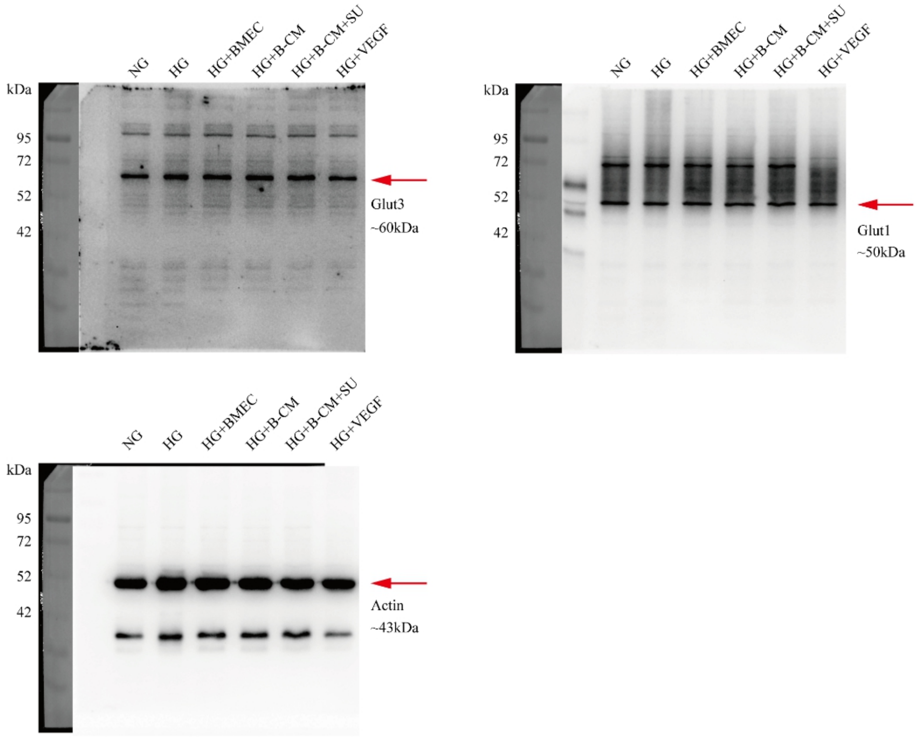

Suppl. Fig. 5A Full-size western blots corresponding to the blots shown in Fig.5A. Samples loaded on the blot correspond to: NG, neurons cultured alone and treated with 25 mM glucose; HG, neurons cultured alone and treated with 45 mM glucose; HG+BMEC, neurons grown with BMECs in 45 mM glucose; HG+B-CM, neurons treated with B-CM and 45 mM glucose; HG+B-CM+ SU, neurons treated with B-CM, 45 mM glucose and 10 μM SU1498; HG+VEGF, neurons treated with 45 mM glucose and 25 ng/ml VEGF.

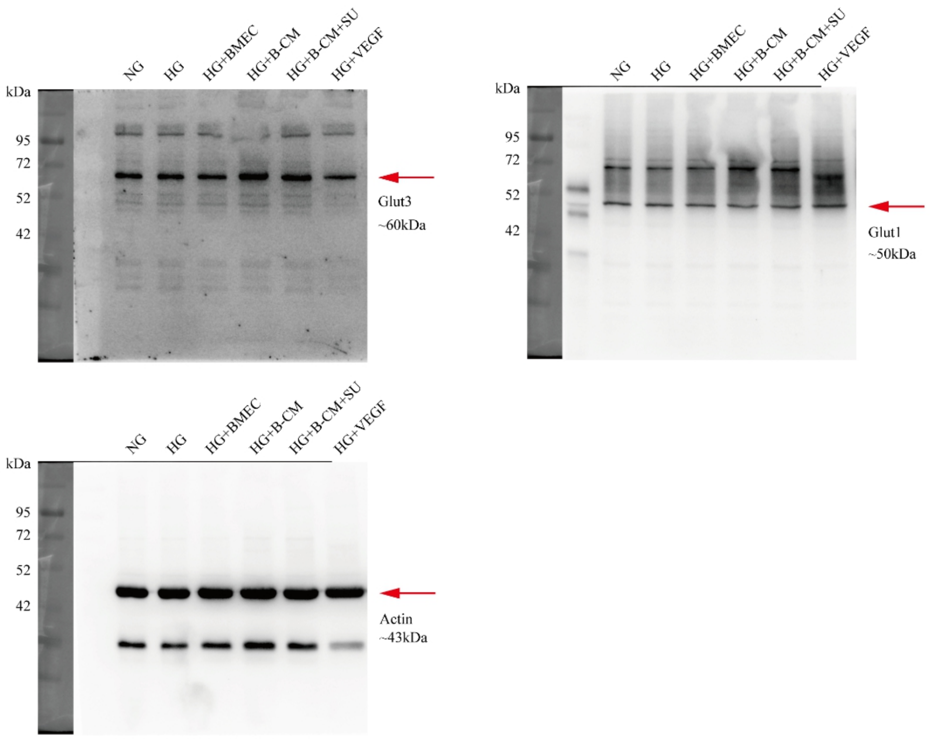

Suppl. Fig. 5B Full-size western blots corresponding to the blots shown in Fig.5B. Samples loaded on the blot correspond to: NG, neurons cultured alone and treated with 25 mM glucose; HG, neurons cultured alone and treated with 45 mM glucose; HG+BMEC, neurons grown with BMECs in 45 mM glucose; HG+B-CM, neurons treated with B-CM and 45 mM glucose; HG+B-CM+ SU, neurons treated with B-CM, 45 mM glucose and 10 μM SU1498; HG+VEGF, neurons treated with 45 mM glucose and 25 ng/ml VEGF.

II Supplementary Tables

Suppl. Table 1 Results of the tests for normality and variance heterogeneity and statistical analysis performed for each experiment (listed by figure number)

Figure number	Group		Normal Distribution?			Homogeneity of variance?					Type of Statistical Analysis
	group	*n*	Yes/No	W	*P*	Yes/No	L	DFn	Dfd	*P*	
fig.1C-a	NG	25	Yes	0.944	0.180	No	5.364	5	144	<0.001	Non-parametric
	HG	25	Yes	0.928	0.080						
	HG+BMEC	25	Yes	0.946	0.204						
	HG+B-CM	25	No	0.869	0.004						
	HG+B-CM+SU	25	No	0.917	0.044						
	NG+SU	25	Yes	0.952	0.284						
fig.1C-b (20μm)	NG	25	Yes	0.967	0.560	Yes	1.722	5	144	0.133	Parametric
	HG	25	Yes	0.925	0.068						
	HG+BMEC	25	Yes	0.924	0.062						
	HG+B-CM	25	Yes	0.934	0.109						
	HG+B-CM+SU	25	Yes	0.946	0.208						
	NG+SU	25	Yes	0.941	0.157						
fig.1C-b (30μm)	NG	25	Yes	0.950	0.245	Yes	2.258	5	144	0.052	Non-parametric
	HG	25	No	0.904	0.022						
	HG+BMEC	25	Yes	0.935	0.114						
	HG+B-CM	25	Yes	0.961	0.437						
	HG+B-CM+SU	25	No	0.895	0.014						
	NG+SU	25	Yes	0.947	0.219						
fig.1C-b (40μm)	NG	25	No	0.893	0.013	No	5.201	5	144	<0.001	Non-parametric
	HG	25	No	0.800	0.000						
	HG+BMEC	25	Yes	0.939	0.140						
	HG+B-CM	25	Yes	0.940	0.147						
	HG+B-CM+SU	25	No	0.829	0.001						
	NG+SU	25	No	0.912	0.033						
fig.1C-b (50μm)	NG	25	No	0.850	0.002	No	5.487	5	144	<0.001	Non-parametric
	HG	25	No	0.562	<0.0001						
	HG+BMEC	25	No	0.894	0.014						
	HG+B-CM	25	Yes	0.933	0.102						
	HG+B-CM+SU	25	No	0.756	<0.0001						
	NG+SU	25	No	0.831	0.001						
fig.1C-c	NG	25	Yes	0.971	0.665	No	4.330	5	144	0.001	Non-parametric
	HG	25	Yes	0.973	0.708						
	HG+BMEC	25	Yes	0.961	0.438						
	HG+B-CM	25	Yes	0.981	0.907						
	HG+B-CM+SU	25	Yes	0.923	0.060						
	NG+SU	25	Yes	0.946	0.198						

fig.1D-a	NG	25	Yes	0.936	0.121	No	7.576	5	144	<0.0001	Non-parametric
	HG	25	Yes	0.956	0.333						
	HG+BMEC	25	No	0.891	0.012						
	HG+B-CM	25	No	0.905	0.024						
	HG+B-CM+SU	25	Yes	0.970	0.634						
	NG+SU	25	Yes	0.934	0.108						
fig.1D-b (20μm)	NG	25	Yes	0.922	0.057	No	2.473	5	144	0.035	Non-parametric
	HG	25	Yes	0.954	0.307						
	HG+BMEC	25	Yes	0.955	0.322						
	HG+B-CM	25	Yes	0.945	0.191						
	HG+B-CM+SU	25	Yes	0.923	0.061						
	NG+SU	25	Yes	0.949	0.233						
fig.1D-b (30μm)	NG	25	Yes	0.924	0.062	Yes	0.543	5	144	0.743	Non-parametric
	HG	25	Yes	0.922	0.058						
	HG+BMEC	25	No	0.913	0.036						
	HG+B-CM	25	Yes	0.950	0.249						
	HG+B-CM+SU	25	Yes	0.960	0.421						
	NG+SU	25	Yes	0.950	0.248						
fig.1D-b (40μm)	NG	25	Yes	0.952	0.283	No	5.481	5	144	<0.001	Non-parametric
	HG	25	No	0.901	0.019						
	HG+BMEC	25	Yes	0.938	0.134						
	HG+B-CM	25	Yes	0.944	0.179						
	HG+B-CM+SU	25	No	0.883	0.008						
	NG+SU	25	Yes	0.941	0.158						
fig.1D-b (50μm)	NG	25	Yes	0.920	0.052	No	4.727	5	144	<0.001	Non-parametric
	HG	25	No	0.811	0.000						
	HG+BMEC	25	Yes	0.933	0.102						
	HG+B-CM	25	Yes	0.944	0.186						
	HG+B-CM+SU	25	No	0.847	0.002						
	NG+SU	25	Yes	0.925	0.068						
fig.1D-b (60μm)	NG	25	No	0.768	<0.0001	No	6.975	5	144	<0.0001	Non-parametric
	HG	25	No	0.752	<0.0001						
	HG+BMEC	25	No	0.832	0.001						
	HG+B-CM	25	Yes	0.939	0.142						
	HG+B-CM+SU	25	No	0.752	<0.0001						
	NG+SU	25	No	0.861	0.003						
fig.1D-b (70μm)	NG	25	No	0.895	0.014	No	7.086	5	144	<0.0001	Non-parametric
	HG	25	No	0.671	<0.0001						
	HG+BMEC	25	No	0.898	0.017						
	HG+B-CM	25	Yes	0.921	0.054						
	HG+B-CM+SU	25	No	0.605	<0.0001						
	NG+SU	25	No	0.761	<0.0001						
fig.1D-b (80μm)	NG	25	No	0.828	0.001	No	15.543	5	144	<0.0001	Non-parametric
	HG	25	No	0.445	<0.0001						

	HG+BMEC	25	No	0.858	0.003						
	HG+B-CM	25	No	0.900	0.018						
	HG+B-CM+SU	25	No	0.560	<0.0001						
	NG+SU	25	No	0.680	<0.0001						
fig.1D-b (90μm)	NG	25	No	0.805	0.000	No	13.472	5	144	<0.0001	Non-parametric
	HG	25	No	0.384	<0.0001						
	HG+BMEC	25	No	0.842	0.001						
	HG+B-CM	25	No	0.817	0.000						
	HG+B-CM+SU	25	No	0.392	<0.0001						
	NG+SU	25	No	0.569	<0.0001						
fig.1D-c	NG	25	Yes	0.942	0.168	No	6.047	5	144	<0.0001	Non-parametric
	HG	25	Yes	0.972	0.691						
	HG+BMEC	25	Yes	0.921	0.054						
	HG+B-CM	25	No	0.898	0.016						
	HG+B-CM+SU	25	Yes	0.961	0.437						
	NG+SU	25	Yes	0.989	0.992						
fig.2C-b	NG	5	Yes	0.899	0.405	Yes	0.675	4	20	0.617	Parametric
	HG	5	Yes	0.915	0.495						
	HG+BMEC	5	Yes	0.953	0.759						
	HG+B-CM	5	Yes	0.928	0.579						
	HG+B-CM+SU	5	Yes	0.938	0.654						
fig.2C-c	NG	5	Yes	0.945	0.699	Yes	0.134	4	20	0.968	Parametric
	HG	5	Yes	0.884	0.330						
	HG+BMEC	5	Yes	0.958	0.795						
	HG+B-CM	5	Yes	0.965	0.843						
	HG+B-CM+SU	5	Yes	0.820	0.118						
fig.2D-b	NG	5	Yes	0.989	0.977	Yes	0.301	4	20	0.874	Parametric
	HG	5	Yes	0.942	0.679						
	HG+BMEC	5	Yes	0.974	0.903						
	HG+B-CM	5	Yes	0.983	0.950						
	HG+B-CM+SU	5	Yes	0.815	0.107						
fig.2D-c	NG	5	Yes	0.908	0.453	Yes	0.481	4	20	0.749	Parametric
	HG	5	Yes	0.894	0.379						
	HG+BMEC	5	Yes	0.793	0.071						
	HG+B-CM	5	Yes	0.888	0.346						
	HG+B-CM+SU	5	Yes	0.937	0.647						
fig.3C-a	NG	25	Yes	0.945	0.194	No	22.020	2	72	<0.0001	Non-parametric
	HG	25	Yes	0.935	0.112						
	HG+VEGF	25	No	0.906	0.024						
fig.3C-b (20μm)	NG	25	No	0.897	0.016	Yes	1.485	2	72	0.233	Non-parametric
	HG	25	Yes	0.926	0.069						
	HG+VEGF	25	Yes	0.923	0.060						
fig.3C-b (30μm)	NG	25	Yes	0.936	0.121	No	9.364	2	72	<0.001	Non-parametric
	HG	25	No	0.907	0.027						

	HG+VEGF	25	No	0.905	0.024						
	NG	25	Yes	0.927	0.074						
fig.3C-b (40μm)	HG	25	No	0.763	<0.0001	No	17.786	2	72	<0.0001	Non-parametric
	HG+VEGF	25	No	0.896	0.015						
	NG	25	No	0.888	0.010						
fig.3C-b (50μm)	HG	25	No	0.493	<0.0001	No	17.387	2	72	<0.0001	Non-parametric
	HG+VEGF	25	No	0.901	0.020						
	NG	25	No	0.794	0.000						
fig.3C-b (60μm)	HG	25	No	0.203	<0.0001	No	33.301	2	72	<0.0001	Non-parametric
	HG+VEGF	25	No	0.793	0.000						
	NG	25	Yes	0.928	0.078						
fig.3C-c	HG	25	Yes	0.952	0.271	No	15.452	2	72	<0.0001	Non-parametric
	HG+VEGF	25	No	0.909	0.029						
	NG	25	Yes	0.938	0.131						
fig.3D-a	HG	25	Yes	0.951	0.263	Yes	2.080	2	72	0.132	Non-parametric
	HG+VEGF	25	No	0.895	0.014						
	NG	25	Yes	0.960	0.412						
fig.3D-b (20μm)	HG	25	Yes	0.951	0.266	Yes	2.749	2	72	0.071	Parametric
	HG+VEGF	25	Yes	0.957	0.355						
	NG	25	Yes	0.957	0.363						
fig.3D-b (30μm)	HG	25	No	0.881	0.007	No	3.242	2	72	0.045	Non-parametric
	HG+VEGF	25	Yes	0.951	0.262						
	NG	25	Yes	0.944	0.185						
fig.3D-b (40μm)	HG	25	No	0.873	0.005	Yes	1.527	2	72	0.224	Non-parametric
	HG+VEGF	25	Yes	0.936	0.121						
	NG	25	Yes	0.962	0.446						
fig.3D-b (50μm)	HG	25	No	0.862	0.003	No	8.419	2	72	<0.001	Non-parametric
	HG+VEGF	25	No	0.903	0.021						
	NG	25	No	0.901	0.019						
fig.3D-b (60μm)	HG	25	No	0.812	0.000	No	3.515	2	72	0.035	Non-parametric
	HG+VEGF	25	No	0.830	0.001						
	NG	25	Yes	0.981	0.896						
fig.3D-c	HG	25	Yes	0.958	0.383	No	3.324	2	72	0.047	Non-parametric
	HG+VEGF	25	Yes	0.972	0.683						
	NG	5	Yes	0.899	0.403						
fig.4C-b	HG	5	Yes	0.904	0.430	Yes	1.744	2	12	0.216	Parametric
	HG+VEGF	5	Yes	0.870	0.267						
	NG	5	Yes	0.918	0.520						
fig.4C-c	HG	5	Yes	0.964	0.832	No	3.964	2	12	0.048	Non-parametric
	HG+VEGF	5	Yes	0.865	0.246						
	NG	5	Yes	0.958	0.792						
fig.4D-b	HG	5	Yes	0.871	0.270	Yes	2.660	2	12	0.111	Parametric
	HG+VEGF	5	Yes	0.914	0.489						
fig4.D-c	NG	5	Yes	0.965	0.845	Yes	0.556	2	12	0.588	Parametric

	HG	5	Yes	0.948	0.726						
	HG+VEGF	5	Yes	0.967	0.856						
fig.5A-b	NG	5	Yes	0.970	0.873	Yes	0.245	5	24	0.938	Parametric
	HG	5	Yes	0.827	0.131						
	HG+BMEC	5	Yes	0.868	0.257						
	HG+B-CM	5	Yes	0.800	0.080						
	HG+B-CM+SU	5	Yes	0.940	0.664						
	HG+VEGF	5	Yes	0.864	0.242						
fig.5A-c	NG	5	No	0.774	0.049	Yes	0.176	5	24	0.969	Non-parametric
	HG	5	Yes	0.972	0.885						
	HG+BMEC	5	Yes	0.925	0.564						
	HG+B-CM	5	Yes	0.922	0.540						
	HG+B-CM+SU	5	Yes	0.902	0.419						
	HG+VEGF	5	Yes	0.903	0.426						
fig.5B-b	NG	5	Yes	0.986	0.962	Yes	0.325	5	24	0.893	Parametric
	HG	5	Yes	0.973	0.893						
	HG+BMEC	5	Yes	0.962	0.820						
	HG+B-CM	5	Yes	0.951	0.741						
	HG+B-CM+SU	5	Yes	0.943	0.687						
	HG+VEGF	5	Yes	0.955	0.771						
fig.5B-c	NG	5	Yes	0.890	0.354	Yes	0.042	5	24	0.999	Parametric
	HG	5	Yes	0.954	0.766						
	HG+BMEC	5	Yes	0.927	0.573						
	HG+B-CM	5	Yes	0.895	0.382						
	HG+B-CM+SU	5	Yes	0.890	0.356						
	HG+VEGF	5	Yes	0.832	0.145						
fig.5D	NG	5	Yes	0.977	0.918	Yes	1.015	5	24	0.431	Parametric
	HG	5	Yes	0.857	0.217						
	HG+BMEC	5	Yes	0.908	0.454						
	HG+B-CM	5	Yes	0.893	0.371						
	HG+B-CM+SU	5	Yes	0.941	0.672						
	HG+VEGF	5	Yes	0.958	0.797						
fig.5E	NG	5	Yes	0.907	0.448	Yes	0.878	5	24	0.511	Parametric
	HG	5	Yes	0.942	0.683						
	HG+BMEC	5	Yes	0.938	0.649						
	HG+B-CM	5	Yes	0.902	0.419						
	HG+B-CM+SU	5	Yes	0.989	0.976						
	HG+VEGF	5	Yes	0.874	0.284						

III Supplementary others

Antibody validation

Anti-PSD95 antibody (Mouse monoclonal to PSD95), Sigma-Aldrich, catalog #: MAB1596, and the antibody was validated by using negative control: DIV3 rat hippocampal neurons (PMID: 25498153).

Anti-Map2 antibody (Mouse monoclonal to Map2), Sigma–Aldrich, catalog #: M9942, and the antibody was validated by using Sprague Dawley rats hippocampus after traumatic brain injury (PMID: 26903822).

Anti-Synapsin I antibody (Rabbit monoclonal to Synapsin I), abcam, catalog #: ab254349, and the antibody was validated by using negative control: mouse lung (PMID: 9539796).

Anti-Map2 antibody (Rabbit polyclonal to MAP2), abcam, catalog #: ab32454, and the antibody was validated by company using mouse brain (day 0) tissue lysate, please refer to themanufacturer's description: https://www.abcam.cn/map2-antibody-neuronal-marker-ab32454.html.

Anti-GLUT1 antibody (Mouse monoclonal to GLUT1), Santa Cruz Biotechnology, catalog #: sc-377228, the antibody was validated by using GLUT1-KD Hep-2 cells (PMID: 30464533).

Anti-GLUT3 antibody (Rabbit polyclonal to GLUT3), Sigma–Aldrich, catalog #: 400062, the antibody was validated by using LRP1-KD neurons (PMID: 25855193).
